# Morphological and Genetic Variation along a North-to-South Transect in *Stipa purpurea*, a Dominant Grass on the Qinghai-Tibetan Plateau: Implications for Response to Climate Change

**DOI:** 10.1371/journal.pone.0161972

**Published:** 2016-08-31

**Authors:** Wensheng Liu, Yao Zhao, Jianling You, Danhui Qi, Yin Zhou, Jiakuan Chen, Zhiping Song

**Affiliations:** 1 The Ministry of Education Key Laboratory for Biodiversity Science and Ecological Engineering, Institute of Biodiversity Science, Fudan University, Shanghai, China; 2 College of Environment Science and Engineering, Southwest Forestry University, Kunming, China; 3 College of Life Science and Technology, Central South University of Forestry and Technology, Changsha, China; Shandong University, CHINA

## Abstract

Estimating the potential of species to cope with rapid environmental climatic modifications is of vital importance for determining their future viability and conservation. The variation between existing populations along a climatic gradient may predict how a species will respond to future climate change. *Stipa purpurea* is a dominant grass species in the alpine steppe and meadow of the Qinghai-Tibetan Plateau (QTP). Ecological niche modelling was applied to *S*. *purpurea*, and its distribution was found to be most strongly correlated with the annual precipitation and the mean temperature of the warmest quarter. We established a north-to-south transect over 2000 km long on the QTP reflecting the gradients of temperature and precipitation, and then we estimated the morphological by sampling fruited tussocks and genetic divergence by using 11 microsatellite markers between 20 populations along the transect. Reproductive traits (the number of seeds and reproductive shoots), the reproductive-vegetative growth ratio and the length of roots in the *S*. *purpurea* populations varied significantly with climate variables. *S*. *purpurea* has high genetic diversity (*He* = 0.585), a large effective population size (*Ne* >1,000), and a considerable level of gene flow between populations. The *S*. *purpurea* populations have a mosaic genetic structure: some distant populations (over 1000 km apart) clustered genetically, whereas closer populations (< 100 km apart) had diverged significantly, suggesting local adaptation. Asymmetrical long-distance inter-population gene flow occurs along the sampling transect and might be mediated by seed dispersal via migratory herbivores, such as the chiru (*Pantholops hodgsonii*). These findings suggest that population performance variation and gene flow both facilitate the response of *S*. *purpurea* to climate change.

## Introduction

Continuous climate change has a major impact on population persistence and species distribution [1]. Estimating the potential that species have to cope with rapid environmental modifications is of vital importance for determining their future viability and developing effective long-term conservation strategies [2, 3]. Organisms can respond to environmental changes by shifting their range or by adapting. Although most plants cannot migrate as rapidly as animals, they may alter their life history strategy, particularly their reproductive strategy, to accommodate local environmental changes. Short-term changes in traits might be due to phenotypic plasticity rather than genetic changes [4]. Phenotypic plasticity enables species to survive rapidly changing local environmental conditions and therefore persist for a longer period of time, during which evolutionary adaptation may occur [5–8]. On a longer time scale, there is the possibility that morphological modifications will occur through natural selection in response to environmental conditions, resulting in local adaptation and maintaining species persistence and integrity and then adaptive population divergence [2, 3, 9–10].

Population divergence results from a delicate balance between the opposing forces of gene flow and diversification via environmentally-generated heterogeneous selection [11, 12]. When gene flow is restricted, specialised genotypes can be maintained in isolated populations, favouring local adaptation; when gene flow is high, it can create a flood of maladaptive nonlocal genes, thereby resulting in “gene swamping,” and local adaptation vanishes [13]. Local adaptive divergence among populations can occur even within the range of gene flow [3, 14]; at intermediate levels, gene flow may maximize local adaptation and thus increase population divergence [13]. The extent to which gene flow have negative or positive effects on adaptation depends on how patterns of gene flow relate to environmental variation. If gene flow is high between populations from different environments, adaptation to local environments may be stalled [12]; if gene flow occurred among similar environments, it might facilitate local adaptation by both increasing population size and introducing new alleles that are locally beneficial [15]. Clearly, estimating the level of gene flow and its effects on population divergence provides important insights into how species respond to continuing climate change.

Although numerous studies of population divergence have been published, models incorporating phenotypic variation, genetic divergence and gene flow combined with climatic variables have proven inadequate to thoroughly understand the response of plant species to ongoing climate change [16]. Phenotypic variation (especially plasticity) and the adaptive divergence of plant species should ideally be characterized using common garden trials. However, it is very difficult to establish common gardens in an area with harsh environmental conditions, such as the Qinghai-Tibetan Plateau (QTP), with a mean altitude over 4000m. An alternative approach is to estimate the variation in biomass allocation among natural populations along an environmental gradient; this may allow us to detect signs of adaptive divergence. Allocation to reproduction is usually size-dependent in plants–that is, it is allometric in the broad sense [17]. For a given genotype, the allometric scaling of reproductive output appears to be a relatively fixed boundary condition [17]. A recent case study confirmed that allometric allocation is genetically determined [18]. Altering allocation in response to environmental changes could therefore reflect an adaptive strategy of plants and have a great impact on the evolution of their life-history [19]. Indeed, recent studies have shown altered allocations along environmental gradients in alpine plant populations [20]. In this study, we examine the morphological and genetic variation of *Stipa purpurea* and assess the role of gene flow and biomass allocation in explaining the population divergence of *S*. *purpurea* along the strong environmental gradients occurring in the QTP to assess population features that may be useful to respond to climate change.

*S*. *purpurea* is a dominant species in the alpine steppe and meadows of the QTP. The QTP is the highest place in the world and is known as “the Third Pole of the Earth.” It is considered a key biodiversity hotspot [21]. Due to the Himalaya Mountains, which block incoming humidity from the Indian Ocean, precipitation decreases from the southeast to the northwest along the QTP, with the average annual precipitation ranging from 20 mm to 600 mm [22]. In addition, although the average elevation of the QTP is around 4000 m, the altitude of the QTP varies from 2000 m (e.g., Chayu County is around 2300 m on average) to over 5000 m (e.g., the Nyainq êntanglha Tanggula Mountains are over 5000 m on average). The variability in conditions has created remarkable environmental heterogeneity on the QTP.

The remarkable environmental gradients on the QTP may lead to strong divergence of *S*. *purpurea* populations and corresponding morphological responses to local habitats. On the other hand, given the continuous distribution of *S*. *purpurea* on the QTP, considerable inter-population gene flow may counteract the population divergence. To test these two hypotheses, we used ecological niche modelling (ENM) to find the most likely climate factors influencing the distribution of *S*. *purpurea* and then established a transect across the QTP based on the results of ENM analyses, along which the genetic and morphological variation of *S*. *purpurea* was surveyed. We aimed to answer the following questions: 1) Is there strong population divergence in *S*. *purpurea*? 2) Is the morphological and genetic variation between populations correlated with climatic variables? and 3) How does gene flow influence population divergence and response to the local environment?

## Material and Methods

### Ethics Statement

No special permits were required for locations and samplings in this study. All samples were collected by researchers with introduction letters of IBSFU (Institute of of Biodiversity Science), Fudan University. The collection was completed with the help of local herdsman

### Species description

**Fig 1:**
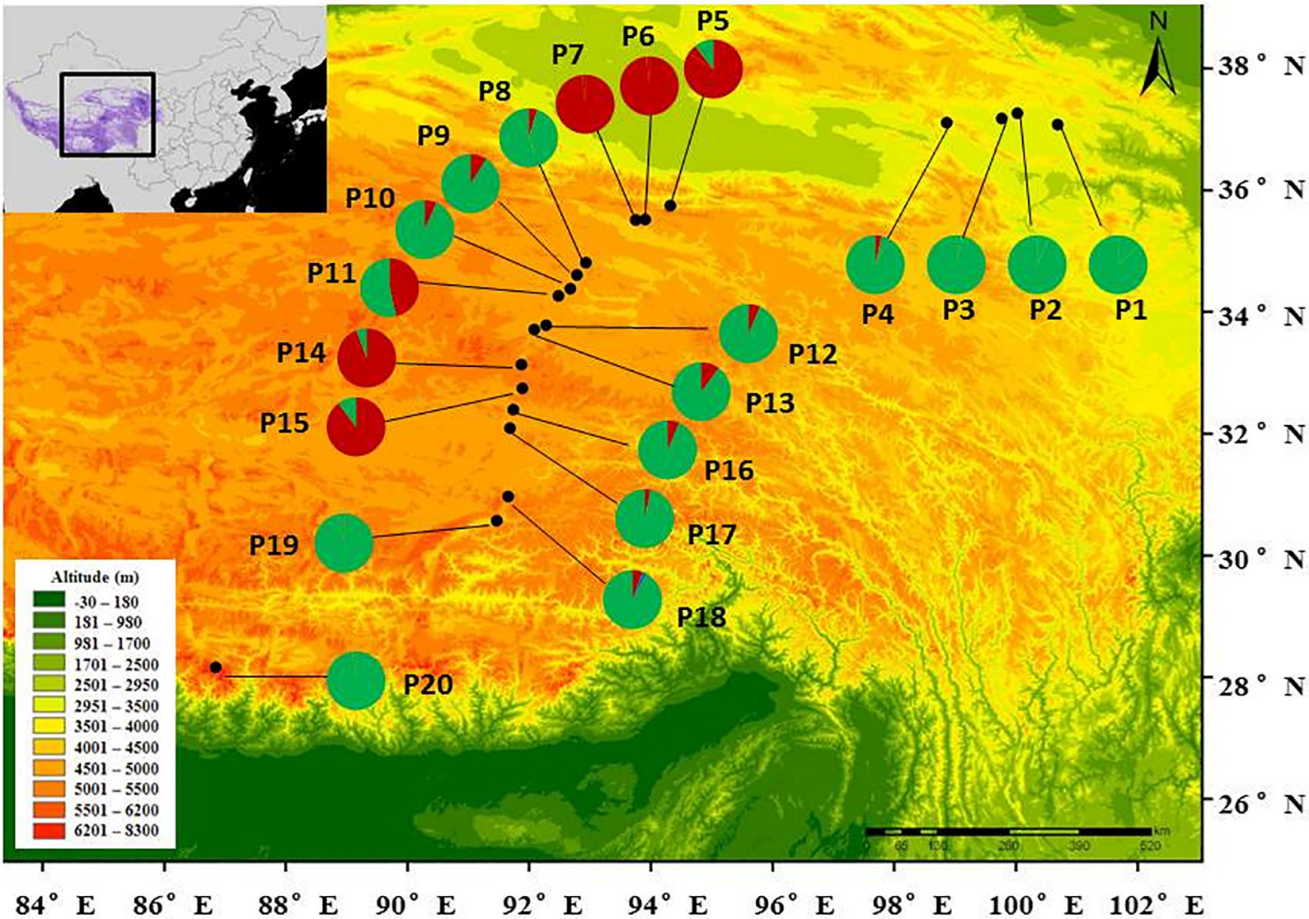
The locations of the 20 sampled *Stipa purpurea* populations on the Qinghai-Tibet Plateau and their genetic divergence. A heatmap of probability of existence generated by Ecological Niche Modelling is shown as a thumbnail image on the left-top. The pie charts indicate the genetic composition for each population based on the result of a representative STRUCTURE run with *K* = 2.

*Stipa purpurea* is endemic to the QTP, Pamirs Plateau and the high mountains of Central Asia [23]. It is a perennial bunchgrass with a mixed mating system and sets seeds from July to October. *S*. *purpurea* can also propagate clonally by tillering [24]. The seed has a long hairy awn that enables it to be dispersed either by wind or zoochorously through attaching to the fur of animals. The species is resistant to cold, drought and high winds, and it can grow well in adverse alpine environments. *S*. *purpurea* plays a paramount role in biodiversity, soil and water conservation [23]. Its distribution covers alpine areas ranging from 1900 to 5150 m in altitude (Fig 1).

### Ecological niche modelling (ENM)

We used a total of 218 *S*. *purpurea* sample locations derived from three sources for ENM. We obtained 69 records from the Chinese Virtual Herbarium (CVH, http://www.cvh.org), 150 records from the Global Biodiversity Information Facility (GBIF, http://www.gbif.org), and 28 records from published population genetics studies [24] and the collections of other authors. Environmental layers of 19 bioclimatic variables (BIO1-19) from the present period (1950–2000) were obtained from the Worldclim version 1.4 website (http://www.worldclim.org). The resolution of all climatic layers was 2.5 arc-minutes (5 km) to ensure compatibility with the resolution of the location data. The study area was defined according to the commonly accepted range of the QTP, between 73.31°E, 104.78°E, 26.00°N and 39.78°N [25].

The current distribution of *S*. *purpurea* was estimated based on the layers for the current climate and the maximum-entropy algorithm (MaxEnt 3.3; [26]). To avoid potential over-parameterization of the model, we based the summary model predictions on 20 replicates using a subsampling method. In each replicate, half of the sample locations were randomly selected as training data and the other half was used as test data. The mean value and standard deviation of the 20 replicates were summarized. The area under the curve (AUC) statistic was obtained to evaluate the general performance of the model [26]. The probability of presence per grid was summarized in a histogram (data not shown), and its average across the study area was calculated. To test the relative importance of each climatic variable, we applied both percentage contribution and permutation approaches. Marginal response curves and single variable response curves were created for the bioclimatic variables. Additional jackknife tests for relative importance and co-linearity among the variables were conducted using the training gain, test gain, and test AUC.

### Sampling design

ENM predicts that the mean temperature of the warmest quarter (*Twarm*) and the annual precipitation (*AP*) are the factors that are most highly correlated with the *S*. *purpurea* distribution. We therefore set up a north-to-south transect over 2000 km to sample *S*. *purpurea* populations across temperature and precipitation gradients on the QTP. A total of 20 populations were sampled along the transect, ranging from 30°N to 37°N in latitude, 91°E to 100°E in longitude and 3200–5100 m in altitude (Fig 1; S1 Table). The *Twarm* and *AP* of the sample sites ranged from 2.68 to 11.07°C and from 164 to 404 mm, respectively. We sampled *S*. *purpurea* populations at the fruiting stage. *S*. *purpurea* has a phalanx-type clonal growth mode, and the ramets of one genet usually form a tussock through clonal reproduction. We considered a tussock as one individual. For each population, 35 individuals were randomly sampled 4 m apart from different tussocks to avoid collecting the same clone of *S*. *purpurea*. Fresh leaf material was desiccated using silica gel, taken to the laboratory and kept at -20°C until DNA extraction.

### Trait measurement

Ten fruited tussocks were randomly collected from each population for trait measurement. The whole tussock was carefully dug up to a depth of 40 cm and then stored in a paper bag after removing the adhered soil. Of the 20 populations sampled for DNA, three did not have a sufficient number of reproductive tussocks to be measured (P9, P16 and P20). Thus, we performed trait measurements on 17 populations in total.

In the laboratory, each plant was separated into roots and shoots. The roots were thoroughly washed and counted (*N*_*root*_). We also recorded the number of vegetative (*N*_*vshoot*_) and reproductive shoots (*N*_*rshoot*_) for each plant. We considered reproductive shoots as spikes and measured the height of the longest spike (*L*_*spike*_). The length of the longest vegetative shoot (*L*_*vshoot*_) and the root (*L*_*root*_) of the plants were measured as well. The seeds were counted and weighted, and the number of seeds (*N*_*seed*_) and the weight of 100 seeds (*M*_*100seed*_) were recorded. The root mass *(M*_*root*_*)*, vegetative shoot mass (*M*_*vshoots*_) and reproductive shoot mass (*M*_*rshoot*_) were measured by weighing after drying in an oven at 60°C to constant weight. The above-ground mass (*M*_*shoot*_) was the sum of the shoot masses (i.e., *M*_*shoot*_ *= M*_*vshoot*_ + *M*_*rshoot*_), and the total mass (*M*_*total*_) was the sum of the shoot and root mass (i.e., *M*_*total*_ *= M*_*shoot*_ + *M*_*root*_). To determine the plant’s resource allocation, the root/shoot ratio (*RSR*) was calculated as *RSR* = *M*_*shoot*_*/M*_*root*_. The allocation of above-ground biomass to reproduction (*RVR*) was expressed as *RVR = M*_*rshoot*_*/M*_*vshoot*_.

### Morphological variation

Descriptive analysis was performed to characterize the morphological variation in the 17 populations for which morphological trait measurements were available. Principal component analysis (PCA) was performed to reduce the trait dataset to sets of interrelated traits using SPSS v19.0.

We determined the proportion of reproductive biomass (R) versus vegetative biomass (V) and shoot biomass (A) versus root biomass (U) using the classical allometric model, R = aVb and A = aUb, fitted as log R = log a + b log V and log A = log a + b log U [27]. The parameters a and b are referred to as the ‘allometric coefficient’ and the ‘allometric exponent,’ respectively. An allometric exponent significantly different from 1.0 indicates an allometric (non-isometric/proportional) relationship. We used standardized major axis (SMA) analysis for fitting the allometric data and estimating the parameters. We tested whether the slope of each population was significantly different from 1.0. All of the SMA analyses were conducted using the software package Standardised Major Axis Tests and Routines (SMATR) [28]. The significance level for determining the heterogeneity slope and a slope difference = 1.0 was *p* < 0.05.

### DNA extraction and PCR assays

DNA was extracted from 25–30 mg of dried leaf material, and a total of 11 SSR loci were amplified as described previously [29] (S2 Table). PCR products were labelled using Fam-, Rox-, and Hex-labelled primers. Alleles were sequenced on an ABI 3730 (ABI) automated sequencer using LIZ 500 as a ladder and then analysed in Genemapper v4.0 (ABI).

### Genetic variation analysis

#### Microsatellite diversity

MICROCHECKER v2.2.3 [30] was used to detect the presence of null alleles and genotyping errors, such as large allele dropout or stuttering, using 1,000 randomizations. Allele frequencies were adjusted for null alleles before use in subsequent analyses. Intra-population genetic variation was estimated using the expected heterozygosity (*He*), observed heterozygosity (*Ho*) and Wright’s fixation index *F* using GenAlEx v6.3 [31]. In addition, the mean effective allele number (*Ae*) and allelic richness (*Ar*) were calculated. Genotypic disequilibrium (LD) between all pairs of loci and the Hardy–Weinberg equilibrium (HWE) were determined for each population, and *p*-values were adjusted with sequential Bonferroni correction for multiple comparisons.

#### Genetic differentiation

We estimated the global *Fst* using GenAlEx v6.3. Partitioning of the total genetic variation within and between populations was further analysed by AMOVA with 1,000 permutations using Arlequin v3.0. The genetic structure was explored using STRUCTURE v2.3.3. The program was given no prior information on ancestral populations and run 10 times for each value of *K* ancestral populations, with *K* varying from 1 to 20, under the admixture model with uncorrelated allele frequencies using 100,000 Markov Chain Monte Carlo iterations and a burn-in of 50,000 iterations. We inferred *K* using the ad hoc statistic Δ*K* [32]. Nei’s genetic distance matrices for all pairs of populations were estimated using MSA v3.0 [33] with 1,000 bootstraps. An unrooted neighbour-joining tree was then constructed using PHYLIP v3.6. The Mantel test was applied to detect the effect of isolation by distance (IBD) on population differentiation. The pairwise geographical distance (*GGD*) matrix and genetic distance (pairwise *Fst/(1-Fst)*) were generated in GenAlEx v6.3 and the Mantel test was performed with 1,000 bootstraps.

#### Spatial analysis of genetic data

We first used a spatial autocorrelation according to the method Peakall and Smouse’s [31] to determine the spatial distribution of gene flow in *S*. *purpurea* populations at the landscape scale based on a frequency-weighted average over all alleles and loci and on the correlation coefficients of genotypes in relation to their spatial distance. The distance class was set to 50 km, and the autocorrelations were performed using GenAlEx v6.3 with 1,000 permutations.

#### Gene flow

To assess the long-term gene flow and the effective population size of *S*. *purpurea*, we applied a coalescent-based approach using the Bayesian and maximum likelihood inference methods implemented in MIGRATE [34] to estimate pairwise migration rates (*M*). MIGRATE jointly estimates the effective population size *ϑ* (4*Ne μ*) and asymmetric gene flow *M* (*m/μ*) between pairs of populations over a long period of time (~4*Ne* generations). Three runs were conducted. First, two shorter runs were performed (10 short chains of 10,000 were sampled and 500 were recorded, then three final chains of 100,000 were sampled and 5,000 were recorded) and used to verify that the MCMC had estimated the parameters correctly. Then, a final long run was performed (10 short chains of 10,000 were sampled and 500 were recorded, then three final chains of 500,000 were sampled and 25,000 were recorded), and *M* values from this final run are reported. The initial run used an estimate of the *Fst* as a starting parameter to calculate *ϑ*, *Ne* and *M*. Each subsequent run used the Maximum-Likelihood (ML) estimates from the previous run as new starting parameters.

BAYESASS [35] was used to estimate contemporary gene flow. BAYESASS uses a Bayesian approach and MCMC sampling to generate mc values (*m*, migration rates) that reflect gene flow over the last few generations. For BAYESASS analyses, each run consisted of 3 000 000 iterations with the chain sampled after 2000 iterations. A burn-in of 1 000 000 was used, and D values were adjusted to ensure that 40–60% of the total changes were accepted. As recommended by [36], we performed 10 runs of BAYESASS, each with a different initial seed, and then used a Bayesian deviance measure to determine the run that best fit the data. Each run consisted of 3 000 000 iterations with the chain sampled after 2000 iterations. For the best fit run, we then ran the analysis again using the seed from the best fit run but increased the run length to 30 000 000 iterations. The results of this final run were presented.

Moreover, MIGRATE and BAYESASS were used to estimate the long-term and contemporary asymmetric gene flow between the genetic groups revealed by STRUCTURE in order to assess the migration rates between regions.

### Habitat heterogeneity

We used all 19 bioclimatic variables to infer habitat heterogeneity. The geographical information for each population was collected with a GPS navigator when sampling. Given the high degree of cross-correlation between climatic variables, PCA was used to reduce the dimensionality of the climatic data set.

To test for correlations between morphological, geographical, and genetic distances among populations, Mantel tests were performed for the 17 populations with trait measurements. Morphological distances (*MD*) were obtained by calculating Euclidean distances between standardized values for the morphological variables of the plants collected in the field. If geographical distance (*GGD*) and genetic distance (pairwise *Fst*) were both significantly correlated with *MD*, then partial Mantel tests were performed to evaluate their relative importance to morphological traits. Morphological variation that significantly correlated with the *GGD* or pairwise *Fst* was further analysed using a partial Mantel test between the *MD* and three climatic principal components difference matrices (PC1clim to PC3clim) after controlling for *GGD* or pairwise *Fst*.

For traits significantly associated with climatic variables, we estimated the independent contributions of these variables to morphological variation using stepwise linear regressions. All analyses were performed using SPSS v19.0.

## Results

### Relative importance of climatic variables revealed by ENM

**Fig 2:**
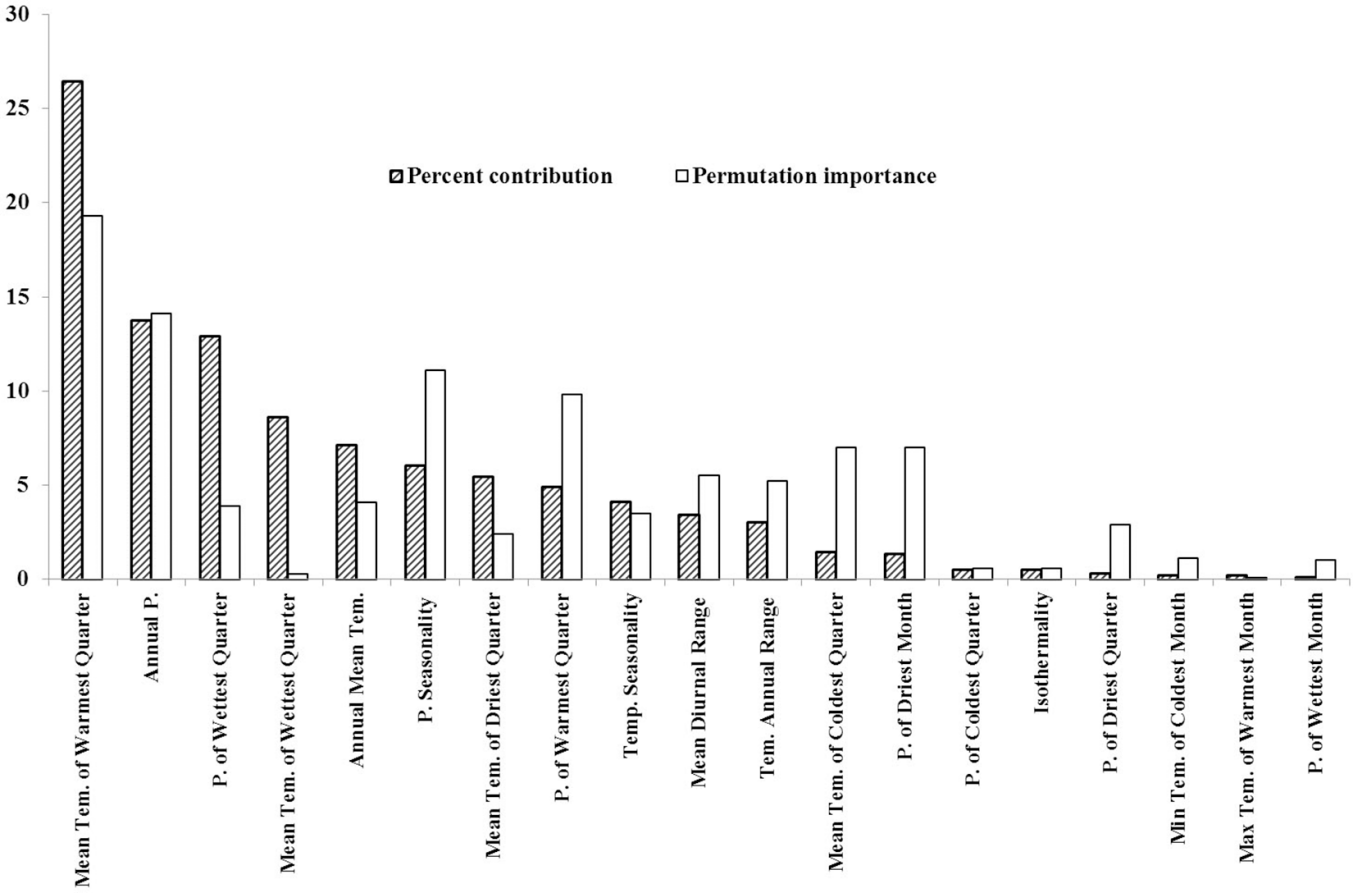
Relative importance of the 19 bioclimatic variables in explaining the distribution of *S*. *purpurea*. The percentage contribution indicates the percentage of variance explained by that particular variable. The permutation importance test indicates the relative importance of bioclimatic variables. (Abbreviations: P., precipitation; Tem., temperature).

The ENM model showed that the mean temperature of the warmest quarter (*Twarm*, bio10), the annual precipitation (*AP*, bio12) and the precipitation of wettest quarter (*Pwet*, bio16) were the most influential climatic variables for *S*. *purpurea* distribution, because they explained about 54% of the variance (Fig 2). In the jackknife evaluation of the training gain, gain test and AUC test, when only a single climatic variable was used, *Twarm*, *AP*, and *Pwet* were also the most important climate factors shaping the *S*. *purpurea* distribution pattern, as were the annual mean temperature (*AT*, bio1) and the mean temperature of wettest quarter (*Twet*, bio8) (S1 Fig; AUC > 0.75).

### Habitat heterogeneity of the sampled populations

From the PCA analysis with varimax rotation of the intercorrelated traits, the first three principal components explained 83.1% of the total variation (Table 1). The first principal component, PC1clim, explained 39.6% of the total variance and was almost perfectly explained by variables related to temperature (bio1, 6, 9, 11). The loading of PC2clim was highly correlated with variables related to variation in temperature (bio7 and 4) and negatively correlated with variables related to precipitation (bio17 and 19). PC3clim was highly correlated with variables related to precipitation (bio12, 16, 18). All 19 climatic variables (S1 Table) were significantly correlated with the altitude, latitude, and longitude of the sampled populations (data not shown). Population P20 had a distinct climate compared with the other populations (S2 Fig).

**Table 1:** Eigen values, percentage of variance explained by each PCA axis, and the first three PC scores of a principle components analysis (PCA) of 19 climatic variables for the 20 sampling locations of *Stipa purpurea*.

	PC1	PC2	PC3
**Eigenvalue**	7.532	5.999	2.251
**Percentage variance explained**	0.396	0.316	0.119
**Eigenvectors**			
Annual Mean Temperature (**bio1**)	0.93	0.275	0.135
Mean Diurnal Range (Mean of monthly (max temp—min temp)) (**bio2**)	-0.424	0.068	0.261
Isothermality ((bio2/bio7)×100) (**bio3**)	-0.157	-0.643	0.269
Temperature Seasonality (S.D.×100) (**bio4**)	-0.158	0.869	-0.266
Max Temperature of Warmest Month (**bio5**)	0.684	0.663	-0.046
Min Temperature of Coldest Month (**bio6**)	0.988	-0.067	0.098
Temperature Annual Range (bio5-bio6) (**bio7**)	-0.173	0.908	-0.161
Mean Temperature of Wettest Quarter (**bio8**)	0.755	0.589	0.018
Mean Temperature of Driest Quarter (**bio9**)	0.939	-0.138	0.25
Mean Temperature of Warmest Quarter (**bio10**)	0.779	0.553	0.014
Mean Temperature of Coldest Quarter (**bio11**)	0.959	-0.111	0.243
Annual Precipitation (**bio12**)	0.216	-0.065	0.954
Precipitation of Wettest Month (**bio13**)	-0.019	-0.306	0.905
Precipitation of Driest Month (**bio14**)	-0.223	-0.222	0.218
Precipitation Seasonality (Coefficient of Variation) (**bio15**)	-0.185	-0.464	0.242
Precipitation of Wettest Quarter (**bio16**)	0.154	-0.166	0.966
Precipitation of Driest Quarter (**bio17**)	-0.088	-0.786	0.32
Precipitation of Warmest Quarter (**bio18**)	0.152	-0.118	0.973
Precipitation of Coldest Quarter (**bio19**)	-0.243	-0.829	0.197

### Phenotypic variation

The morphological traits all differed significantly between populations (S3 Table). The lowest variation between populations was a 2.9-fold difference in *L*_*spike*_, ranging from 9.26 cm to 26.67 cm, whereas *N*_*root*_ showed a 15-fold difference, ranging from 31 to 472.

A PCA on the 14 morphological traits showed that the first three principal components accounted for 75.2% of the total variance (Table 2). PC1, which explained 45.2% of the total variance, was highly correlated with biomass-related traits, such as the number of shoots (*N*_*vshoot*_), the biomass of the shoot (*M*_*vshoot*_), and the total biomass (*M*_*total*_) (S3 Fig). PC2 was highly correlated with reproductive traits, such as the biomass of the reproductive shoot (*M*_*rshoot*_), the biomass of 100 seeds (*M*_*100seed*_), and the reproductive fraction of biomass of the above-ground material (*RVR*), explaining 18.3% of the total variance. PC3 was highly correlated with the height of the plants (*L*_*spike*_, *L*_*vshoot*_) and seed number (*N*_*seed*_), and explained 11.7% of the total variance. A scatterplot of PC1 against PC2 showed that the morphological variations of populations P13 and P18 are distinct (S3 Fig).

**Table 2:** Eigen values, percentage of variance explained by each PCA axis, and the first three PC scores of a principle components analysis (PCA) of 14 morphological traits for *S*. *purpurea* populations.

	PC1	PC2	PC3
**Eigenvalue**	5.431	2.192	1.403
**Percentage variation explained**	45.2%	18.3%	11.7%
**Eigenvectors**			
***N***_***vshoot***_	0.891	-0.137	-0.181
***L***_***vshoot***_	0.588	-0.002	0.666
***M***_***vshoot***_	0.919	0.009	0.214
***N***_***rshoot***_	0.599	0.445	-0.198
***L***_***spike***_	-0.013	0.127	0.878
***M***_***rshoot***_	0.579	0.762	0.224
***N***_***seed***_	-0.170	-0.214	0.634
***M***_***100seed***_	0.223	0.723	-0.189
***N***_***root***_	0.698	-0.025	-0.315
***L***_***root***_	0.756	-0.262	0.000
***M***_***root***_	0.787	0.063	0.182
***M***_***total***_	0.918	0.177	0.194
***RVR***	-0.285	0.898	0.089
***RSR***	0.051	-0.238	-0.016

### Allometric allocation

Only 3 of the 17 S. purpurea populations in which traits were measured showed a significant positive correlation between log *M*_*rshoot*_ and log *M*_*vshoot*_ (S4 Table). P6 and P14 had slopes significantly > 1.0, and the slope of P1 was significantly < 1.0. However, these populations did not show allometric allocation because of extremely low correlation coefficients (*r*) or a violation of the assumption of normality. In addition, there was no sign of allometric allocation between *M*_*shoot*_ and *M*_*root*_ (data not shown).

### Population genetics

#### Allelic diversity

A total of 150 alleles were detected at 11 loci. The effective number of alleles (*Ae*) varied from 2.1 to 3.9 per locus, with an average value of 3.0 (Table 3), and allelic richness (*A*_*r*_) at different loci ranged from 2.7 to 6.1, with an average value of 5.1. No significant departures from linkage equilibrium were detected for any pair of loci in each population, whereas significant deviations from Hardy–Weinberg equilibrium were detected for 10 loci after Bonferroni correction (*p* < 0.05). The *S*. *purpurea* populations had high genetic diversity, with an expected heterozygosity (*He*) ranging from 0.442 to 0.704. The overall inbreeding coefficient (*F*) ranged from -0.288 to 0.026 (Table 3).

**Table 3:** Parameters of genetic diversity of 20 *S*. *purpurea* populations based on 11 microsatellites. Mean values are shown, with the standard error in brackets.

Pop	*N*	*Ae*	*Ar*	*Ho*	*He*	*F*
**P1**	35	3.837 (0.610)	6.058 (1.827)	0.652 (0.059)	0.664 (0.060)	-0.003 (0.032)
**P2**	35	2.764 (0.545)	3.578 (1.078)	0.551 (0.111)	0.492 (0.095)	-0.078 (0.116)
**P3**	35	2.629 (0.250)	4.973 (1.499)	0.608 (0.076)	0.572 (0.064)	-0.059 (0.033)
**P4**	35	3.131 (0.417)	5.393 (1.626)	0.577 (0.089)	0.583 (0.084)	0.026 (0.043)
**P5**	35	3.070 (0.334)	5.615 (1.693)	0.647 (0.059)	0.633 (0.049)	-0.036 (0.051)
**P6**	35	2.260 (0.325)	3.877 (1.169)	0.525 (0.094)	0.461 (0.079)	**-0.132 (0.044)**
**P7**	35	2.115 (0.195)	3.194 (0.963)	0.603 (0.071)	0.495 (0.044)	**-0.216 (0.086)**
**P8**	35	3.642 (0.239)	6.010 (1.812)	0.782 (0.017)	0.726 (0.015)	**-0.095 (0.026)**
**P9**	35	3.374 (0.503)	5.326 (1.606)	0.688 (0.057)	0.658 (0.046)	-0.059 (0.047)
**P10**	35	3.181 (0.502)	5.349 (1.613)	0.631 (0.067)	0.615 (0.067)	-0.049 (0.038)
**P11**	35	3.598 (0.480)	5.811 (1.752)	0.732 (0.091)	0.645 (0.077)	**-0.144 (0.025)**
**P12**	35	3.006 (0.445)	4.445 (1.340)	0.605 (0.083)	0.585 (0.072)	-0.036 (0.040)
**P13**	35	3.945 (0.706)	5.947 (1.793)	0.709 (0.039)	0.704 (0.035)	-0.026 (0.036)
**P14**	35	2.660 (0.356)	4.314 (1.301)	0.629 (0.083)	0.553 (0.068)	**-0.137 (0.054)**
**P15**	35	3.043 (0.428)	4.018 (1.212)	0.717 (0.058)	0.625 (0.045)	**-0.173 (0.082)**
**P16**	35	2.934 (0.396)	4.918 (1.483)	0.603 (0.090)	0.572 (0.082)	-0.070 (0.061)
**P17**	35	2.992 (0.311)	5.285 (1.593)	0.668 (0.080)	0.615 (0.066)	**-0.109 (0.077)**
**P18**	35	2.915 (0.328)	4.865 (1.467)	0.634 (0.088)	0.585 (0.078)	**-0.104 (0.054)**
**P19**	35	2.599 (0.409)	4.004 (1.207)	0.535 (0.118)	0.482 (0.096)	0.008 (0.124)
**P20**	35	2.129 (0.276)	2.681 (0.808)	0.571 (0.113)	0.442 (0.080)	**-0.288 (0.116)**
**Mean**	35	2.991 (0.096)	5.064 (2.390)	0.633 (0.018)	0.585 (0.015)	**-0.087 (0.015)**

*N*, sample size; *Ae*, number of effective alleles; *Ar*, allelic richness; *Ho*, observed heterozygosity; *He*, expected heterozygosity; *F*, fixation index. The *F* values significantly deviated from 0 were shown in bold font.

#### Genetic divergence

**Fig 3:**
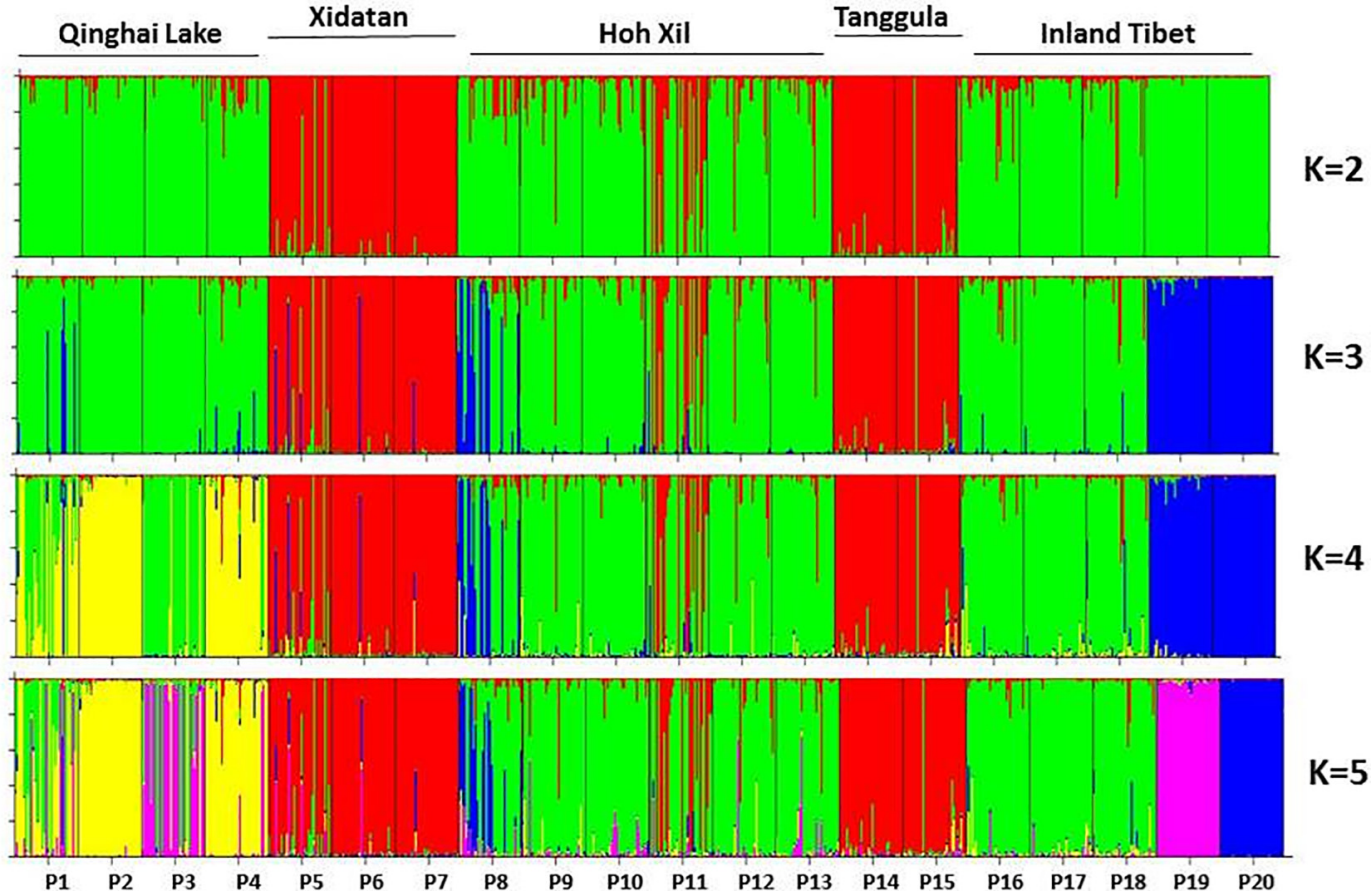
Genetic divergence in the *S*. *purpurea* populations illustrated by STRUCTURE with *K* = 2 to 5. Each line represent the membership coefficients (*Q*) of individual plants. Population codes are indicated below and the sampled geographical regions.are shown above.

**Fig 4:**
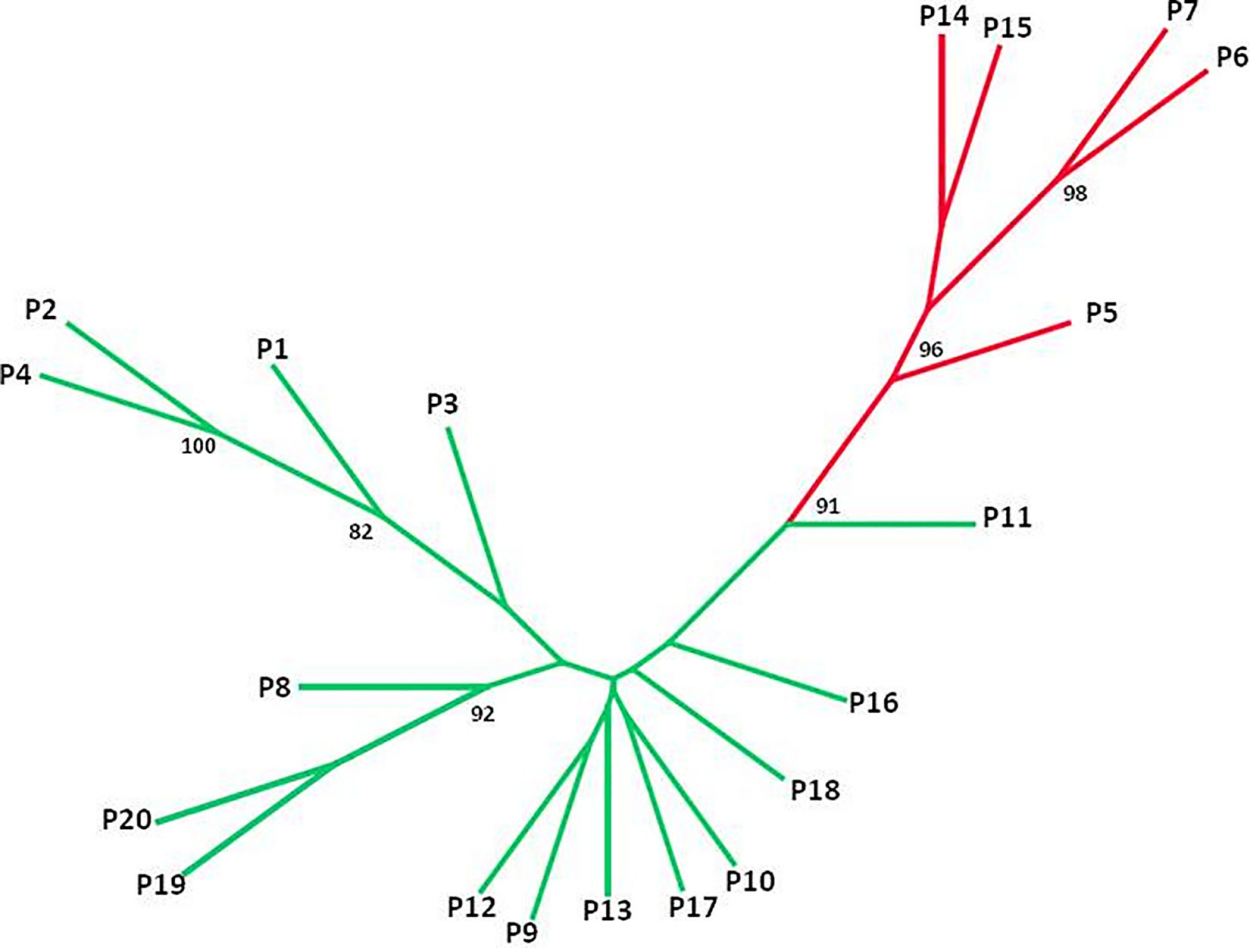
Unrooted neighbor-joining tree of *S*. *purpurea* populations based on Nei's genetic distance. Numbers at nodes are significant bootstrap support percentages from 1,000 replicates on loci. Colors correspond to *K* = 2.

**Fig 5:**
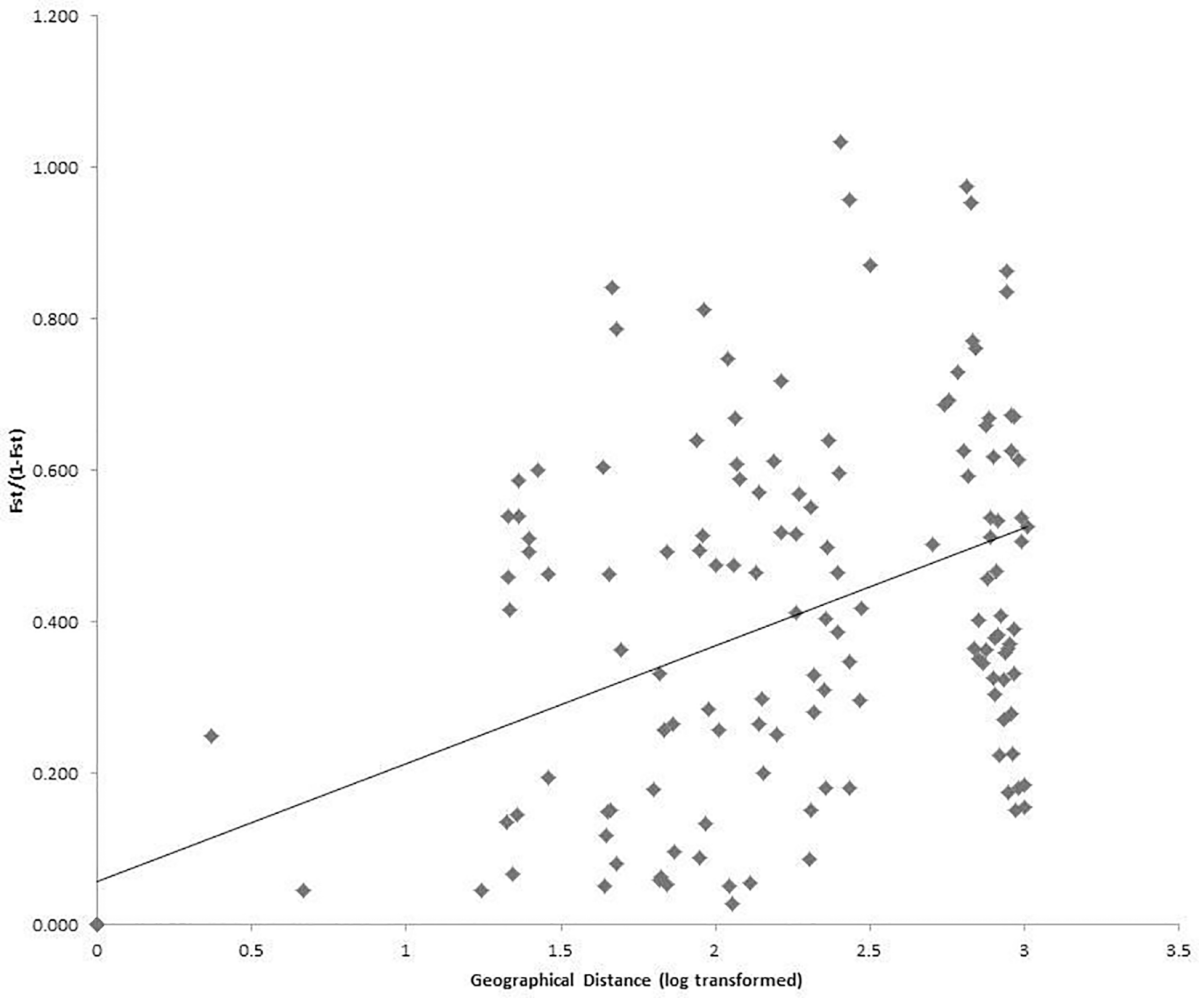
Scatterplot between genetic distance and geographic distance for *Stipa purpurea* populations. Genetic distance is represented by pairwise *F*_*ST*_/(1–*F*_*ST*_) among populations, which is correlated with the geographic distance between pairwise populations. The Pearson’s regression line overlays the scatterplot (Mantel-test, *R*^*2*^ = 0.21, *p* < 0.001).

The average population differentiation coefficient (*Fst*) was 0.198 for the 11 SSR markers (S2 Table). Hierarchical analysis of molecular variance (AMOVA) showed that 66.0% of the total genetic variation existed within populations and 34.0% between populations. In the STRUCTURE analysis, plotting Δ*K* against a range of *K* values revealed the highest peak at *K* = 2, with a lower peak at 5 (S4 Fig). With *K* = 2, the populations from two geographically discontinuous regions (Xidatan [P5, P6 and P7] and the Tanggula Mountains [P14 and P15]) clustered together (Fig 3), showing a mosaic pattern across the sampling transect and forming the following 5 adjacent, divergent groups: 1) Qinghai Lake (QL); 2) Xidatan (XD); 3) Hoh Xil (HX); 4) Tanggula Mountains (TM); and 5) Inland Tibet (IT). At *K* = 5, further subdivisions occurred in the region of Qinghai Lake and between P19 and P20. We also showed the results of STRUCTURE when *K* = 3 & 4. At *K* = 3, the general differentiation pattern of regions along the sampling transect was similar as *K* = 2, while within IT, the population P19 and P20 formed an additional group (Fig 3). And at *K* = 4, subdivisions occurred within QL, the population P1 and P4 separated from another populations (Fig 3). The unrooted NJ tree reinforced the pattern revealed by STRUCTURE (Fig 4). The Mantel test showed significant support for isolation by distance (IBD) in *S*. *purpurea* (*R*^2^ = 0.21, *p* < 0.001) (Fig 5).

#### Gene flow

**Fig 6:**
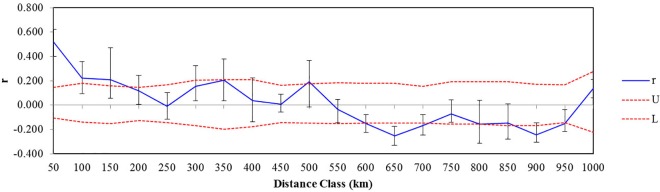
Correlograms showing the genetic correlation per distance class against geographical distance. Dotted lines indicate the 95% C.I. about the null hypothesis of random distribution of genotypes and individuals. The error bars indicate the 95% confidence error bars of *r* as determined by bootstrapping.

The autocorrelation analysis showed that there was significant spatial genetic structure (SGS) up to a distance class of 200 km (Fig 6), indicating gene flow at a landscape scale. Coalescent-based Bayesian estimates by MIGRATE indicated that *S*. *purpurea* populations have relatively large effective population sizes, with *Ne* > 1,000 (S5 Table), and considerable inter-population long-term gene flow (mean *M* = 1.343), which is similar to the estimate based on *Fst* values (*Nm* = 1.184) (S2 Table). The mean contemporary gene flow calculated by BAYESASS was *m* = 0.008.

**Fig 7:**
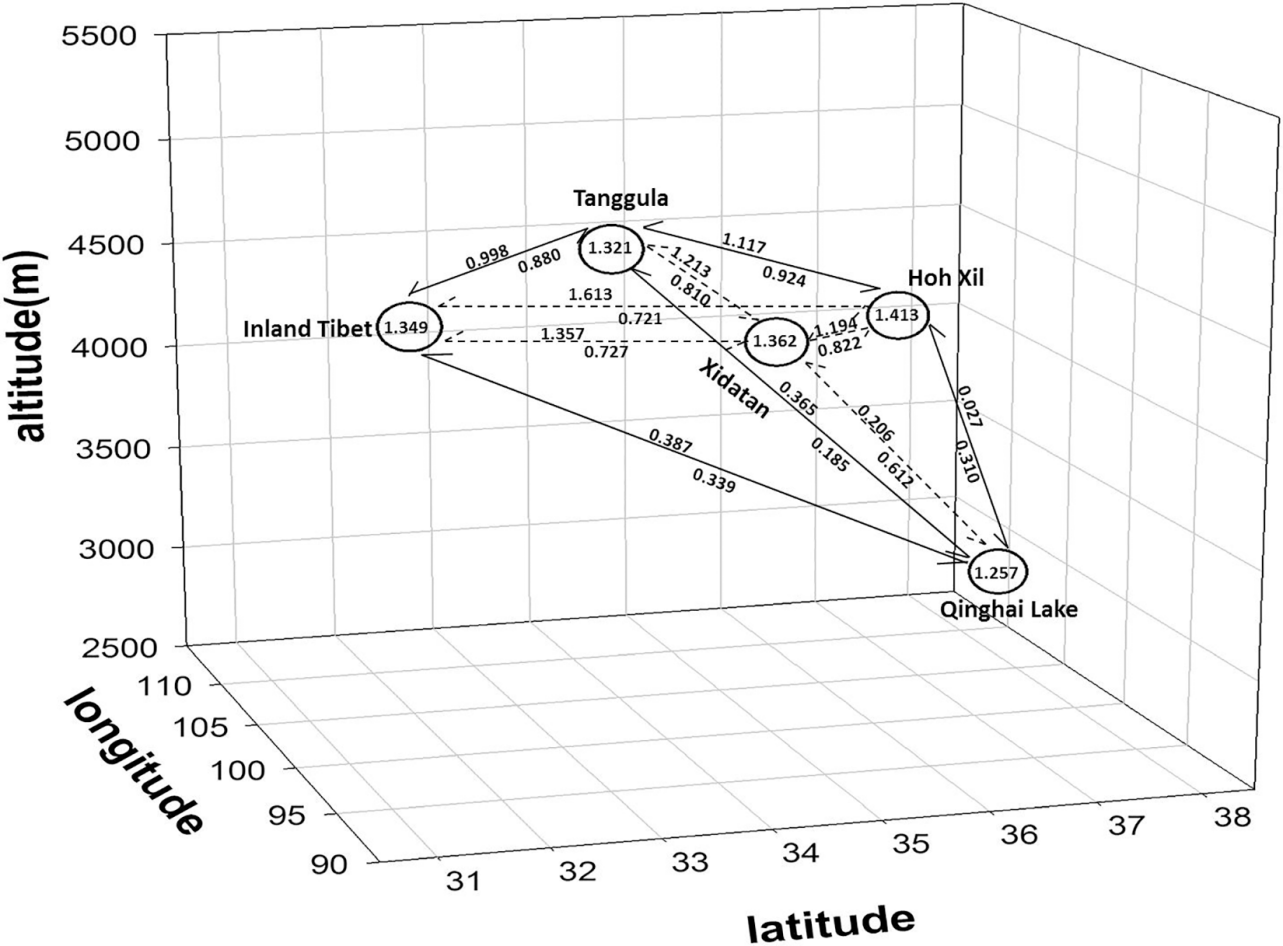
Number of migrants per generation (*M*) between genetically divergent regions. *Nm* was calculated by a Bayesian method implemented in MIGRATE. Numbers in circles indicate *Nm* between populations within a region; numbers above or below the lines are *Nm* between regions; arrows show the direction of gene flow. The regions shown are Inland Tibet (IT), Tanggula Mountains (TM), Xidatan (XD), Hoh Xil (HX), and Qinghai Lake (QL) based on the results of STRUCTURE when *K* = 2 (Fig 3).

According to the results of STRUCTURE, when *K* = 2 the sample map is divided into five regions (Fig 3). Between these regions, the long-term inter-region gene flow (*M*) ranged from 0.027 to 1.613, with the highest migration rate observed from Hoh Xil to the Inland Tibet region (*M* = 1.613) (Fig 7; S6 Table). The estimates of migration rates between Qinghai Lake and the other regions were much lower (*M* <0.612), whereas higher gene flow occurred between the other regions (*M* = 0.723–1.613). Long-term migration between regions was asymmetrical, as shown by the number of migrants (*M*) in Fig 7. The contemporary gene flow (*m*) between regions ranged from 0.0016 to 0.1651 (S7 Table), with a similar trend to the pattern of historical gene flow; and a relatively high level of gene flow also occurred from Hoh Xil to Tanggula.

### Population variation associated with environmental factors

The Mantel tests of morphological distance (*MD)* against geographic distance (*GGD*) between populations revealed a significant effect of isolation by distance on the differentiation of *M*_*vshoot*_, *N*_*rshoot*_, *M*_*rshoot*_, *L*_*root*_, *M*_*total*_ and *N*_*seed*_ (Table 4). Significant correlations between pairwise *MD* and *Fst* were found for *N*_*rshoot*_, *N*_*root*_, *L*_*root*_ and *M*_*total*_ (Table 4). When controlling for either *GGD* or *Fst*, *N*_*rshoot*_ was still significantly correlated with the other matrix, indicating the joint effect of demography and/or population history on this trait and on geographical factors. For *L*_*root*_ and *M*_*total*_, the partial Mantel tests suggested that geographical distribution was more important to the structure of trait variation.

**Table 4:** Mantel tests of the relationship between morphological distances (*MD*) and partial Mantel tests while controlling for pairwise differentiation in neutral genetic markers (*Fst*) or geographic distance (GGD).

*MD*		GGD	*Fst*	GGD|*Fst*	*Fst*|GGD
***M***_***vshoot***_	**r**	**0.191**	0.080	-	-
	**p**	**0.026**	0.352	-	-
***N***_***rshoot***_	**r**	**0.318**	**0.362**	**0.244**	**0.302**
	**p**	**0.000**	**0.000**	**0.004**	**0.000**
***M***_***rshoot***_	**r**	**0.284**	0.019	-	-
	**p**	**0.001**	0.822	-	-
***L***_***root***_	**r**	**0.226**	**0.177**	**0.184**	0.123
	**p**	**0.008**	**0.039**	**0.040**	0.157
***M***_***total***_	**r**	**0.254**	**0.196**	**0.228**	0.068
	**p**	**0.003**	**0.022**	**0.008**	0.434
***N***_***seed***_	**r**	**0.642**	0.073	-	-
	**p**	**0.000**	0.400	-	-
***N***_***root***_	**r**	-0.021	**-0.220**	-	-
	**p**	0.806	**0.010**	-	-

Only the traits significantly correlated with GGD or/and *Fst* are listed. The significant Mantel tests and partial Mantel tests are marked in bold.

When a trait was significantly correlated with *GGD* or *Fst*, the variation across the sampling transect might be due to local climatic variables resulting from a heterogeneous QTP habitat. Partial Mantel tests between *MD* and the three climatic principal components difference matrices (PCclim1 to PCclim3) controlling for *GGD* or *Fst* revealed that *M*_*vshoot*_, *N*_*rshoot*_, *L*_*root*_, *N*_*seed*_ and *M*_*total*_ were significantly influenced by climatic variables (PC1clim & PC2clim; PC3clim did not show a correlation with any *MD*) (Table 5).

**Table 5:** Mantel test between morphological distance (*MD*) and principle components difference matrices of climate variables and partial Mantel tests when controlling for pairwise *Fst* and GGD, respectively. PC3clim in Table 1 showed no correlations with any *MDs*.

***MD***	vs PC1clim		Controlling for *Fst*		Controlling for GGD	
	r	p	r	p	r	p
***M***_***vshoot***_	0.031	0.691	0.003	0.978	-0.061	0.494
***N***_***rshoot***_	**0.242**	**0.003**	0.134	0.100	0.123	0.121
***M***_***rshoot***_	0.007	0.935	0.001	0.991	-0.153	0.087
***N***_***root***_	**-0.186**	**0.035**	-0.117	0.221	**-0.189**	**0.026**
***L***_***root***_	**0.187**	**0.022**	0.135	0.100	0.103	0.229
***M***_***total***_	0.053	0.539	0.004	0.965	-0.073	0.396
***N***_***seed***_	**0.452**	**0.000**	**0.457**	**0.000**	**0.227**	**0.008**
***MD***	vs PC2clim		Controlling for *Fst*		Controlling for GGD	
	r	p	r	p	r	p
***M***_***vshoot***_	**0.361**	**0.000**	**0.357**	**0.000**	**0.317**	**0.000**
***N***_***rshoot***_	**0.395**	**0.000**	**0.309**	**0.000**	**0.262**	**0.002**
***M***_***rshoot***_	**0.220**	**0.009**	**0.226**	**0.009**	0.058	0.522
***N***_***root***_	-0.070	0.444	0.007	0.935	-0.075	0.432
***L***_***root***_	**0.335**	**0.000**	**0.299**	**0.001**	**0.253**	**0.003**
***M***_***total***_	**0.331**	**0.000**	**0.304**	**0.000**	**0.220**	**0.008**
***N***_***seed***_	**0.340**	**0.000**	**0.337**	**0.000**	-0.105	0.278

Further analyses showed that among the traits associated with climate variables (PCclim), *N*_*seed*_ was correlated with annual mean temperature (bio1) and mean temperature of the coldest quarter (bio11), explaining 21.1% and 5.9% of total variance, respectively; *N*_*rshoot*_ was mainly dependent on precipitation during the harsh period of the year (bio17 and bio19), accounting for 3.2% and 10.7% of total variance; and *L*_*root*_ was associated with temperature seasonality (bio4) and precipitation of the driest quarter (bio17), though those only accounted for 4.8% and 4.2% of the total variance, respectively (Table 6). Although *M*_*vshoot*_ and *M*_*total*_ were significantly correlated with PC2clim, they showed no relationship with the main climatic variables in PC2clim.

**Table 6:** Hierarchical partitioning of the independent effects of the nine climatic variables reflecting habitat heterogeneity on morphological traits in 17 natural populations of *S*. *purpurea*.

Climatic variables	Fraction of regression relationship (%) attributable independently to environmental variables for each trait		
	*N*_*seed*_	*N*_*rshoot*_	*L*_*root*_
Annual Mean Temperature **(bio1)**	0.46 (21.10%)	-	-
Temperature Seasonality **(bio4)**	-	-	0.219 (4.80%)
Mean Temperature of Coldest Quarter **(bio11)**	0.06 (5.94%)	-	-
Precipitation of Driest Quarter **(bio17)**	-	0.046 (3.20%)	0.081 (4.20%)
Precipitation of Coldest Quarter **(bio19)**	-	0.327 (10.70%)	-

Non-significant contributions (*p* > 0.05) are omitted. Probabilities are based on bootstrapping with 1000 repetitions.

## Discussion

Plants interact with the changing environment in multiple ways. In this study, we found environmentally associated morphological variation and a mosaic population genetic structure with asymmetrical inter-population gene flow in *S*. *purpurea*, which may indicate an adaptive response to local climatic variables.

### Morphological divergence between populations

The PCA revealed that the variation in *M*_*total*_, *M*_*vshoot*_, *N*_*vshoot*_, *M*_*100seed*_, *M*_*rshoot*_, *M*_*total*_, *L*_*spike*_, and *RVR* (reproductive/vegetative growth ratio) best explained the morphological divergence between populations (Table 2) and seemed to demonstrate that the surveyed natural *S*. *purpurea* populations diverged mainly in plant size. However, we did not detect significant allometric allocation between reproductive and vegetative growth or between the above- and below-ground biomass in most populations (S4 Table). This is not consistent with the results of Guo *et al*. (2012) [20], who found a significant size-dependent resource allocation to reproduction in *Pedicularis* on the QTP along an elevation gradient. Nevertheless, our findings do not preclude the possibility of adaptive divergence between populations. For instance, *RVR* contributed to population variation (Table 2), which suggests a trade-off in reproductive biomass allocation for populations along environmental gradients (PC2clim, a proxy of temperature annual range (bio7), Table 1).

The Mantel tests revealed that most of the variation in traits between populations was correlated with a geographical factor and/or *Fst* (Table 4), and the partial Mantel tests showed that the clear divergence of *M*_*vshoot*_, *N*_*rshoot*_, *L*_*root*_, *N*_*seed*_ and *M*_*total*_ was associated with climatic variables (Table 5), suggesting the possibility of adaptive divergence. The partial Mantel tests further revealed that the corresponding climate variables (Table 6) shaped the divergence patterns of *N*_*seed*_, *N*_*rshoot*_ and *L*_*root*_.

Half of the traits for which we detected evidence of adaptive divergence are related to reproduction (*N*_*rshoot*_, *M*_*rshoot*_, *N*_*seed*_ and *RVR*) and the others involve allocation. These findings demonstrate the importance of modifications to reproductive strategy for plants to survive under different conditions [3]. Furthermore, we found that the number of seeds increased with the temperature and that the number of reproductive shoots increased with an increase in the precipitation of the driest and coldest quarter (Table 6). This is consistent with findings in *S*. *krylovii* [37]. More seeds may imply greater drought tolerance [38], and more reproductive shoots means a higher seed yield and more allocation toward reproduction. Although no significant trade-off between seed number and seed mass was detected, our findings suggest that, as a cold-adapted plant, *S*. *purpurea* may open a high fraction of florets in relatively warm places to increase pollination but may allocate more to reproduction under relatively dry conditions. We found that *L*_*root*_ decreased with an increase in temperature seasonality and the precipitation of the driest quarter (Table 6). This finding indirectly supports the optimal proportion theory–the idea that plants allocate biomass to the organ that acquires the most limiting resource. Under cold and arid conditions, plants are thus apt to allocate more energy to the roots [39]. These findings together suggest that the response of *S*. *purpurea* populations to local habitat might be adaptive. Common garden trials may provide more direct evidence of local adaption to habitat heterogeneity.

### Genetic variation

Genetic variability is required for populations to evolve in response to environmental changes. *S*. *purpurea* is a perennial, wind-pollinated plant; as a dominant grass, it has a relatively complex demographic history and a broad ecological range on the QTP [23]. These biological characteristics could all contribute to the high level of genetic variability observed (*He* = 0.585, Table 3). The high genetic diversity might also be due to the relatively large effective population size (*Ne* > 1,000, S5 Table) and frequent gene flow between populations (mean *Nm* > 1.0, S2 and S6 Tables, Fig 7). Sexual reproduction may also play an important role in maintaining genetic variability. Although *S*. *purpurea* is known to be capable of selfing, the *F* index data (Table 3) suggest that it maintains a high degree of outcrossing, similar to its congeners (e.g., Durka *et al*. 2013 [40]; Hamasha *et al*. 2013 [41]). The high level of genetic variability within populations of *S*. *purpurea* is similar to that of other outcrossing perennial grasses and comparable to that of the perennial and woody species that range across most areas of the Qinghai-Tibetan plateau (e.g., Tibetan poplar, *He* = 0.488 (Shen *et al*. 2014)[42]; Siberian apricot, *He* = 0.774 (Wang *et al*. 2014) [43]).

Significant heterozygote excess was observed in most populations (Table 3). There are several potential causes for this, including a small *Ne*, heterozygote advantage, and self-incompatibility [44]. Heterozygote advantage may be the primary cause for the heterozygote excess. Outcrossed genotypes grow faster and have lower mortality than progeny from selfing; as a result, populations show greater heterozygosity due to the selective loss of homozygous individuals. In addition, greater heterozygosity might be expected in genotypes performing asexual propagation, because they could persist in a population over the course of several sexual generations [44]. This assumption is in line with the view that high heterozygosity could be favourable in long-lived plants that grow in arid zones [43], though more detailed genotyping analysis is needed to address the question thoroughly.

The genetic divergence between *S*. *purpurea* populations is moderate (*Fst* = 0.198). Significant IBD was supported by a Mantel test and the result of autocorrelation analyses (Figs 5 and 6). In this scenario, the populations should show geographically-dependent divergence. However, the *S*. *purpurea* populations do not cluster according to their origins (Fig 1). Some populations (such as P13 and P14, P15 and P16) have significant genetic divergence though they are not geographically distant (Figs 3 and 4), which suggests the isolation of habitats and/or habitat differentiation at a fine-scale, though their habitats are continuous. Moreover, populations from the Xidatan region clustered with populations from the Tanggula Mountains rather than the nearer populations, whereas populations from Qinghai Lake, Hoh Xil, and the Inland Tibet region formed one genetic group and showed a mosaic genetic structure at the landscape scale. Long-distance gene flow and/or local adaptation to similar habitats are likely explanations for the close genetic relationships between distant populations. For example, Xidatan and the Tanggula Mountains have similarly harsh environments, so local adaptation could make populations in the two regions similar regardless of the homogenizing effects of gene flow with nearby populations [14, 45]. Such a pattern may be explained by environmental variation rather than geographic distance, suggesting a role for habitat heterogeneity in the genetic divergence of populations. Thus, when interpreting the current distribution patterns of genetic variation or attempting to predict the likely responses to climate change, it is critical to include relevant information about the natural history of plants rather than relying on information about gene flow alone. More detailed sampling is required to test this hypothesis.

### Long-distance asymmetrical gene flow

Disentangling the impact of dispersal and seed establishment as causes of gene flow is essential for climate change research. Although the abiotic factors that drive seed and pollen dispersal, such as topography and local wind dynamics, may remain constant in a changing environment, zoochory may be altered by changes in the environment and also provides plants with opportunities to find suitable new habitats. Our study revealed that gene flow was not restricted to within populations, as was expected (Liu *et al*. 2009, *Nm* = 0.78 [24]); instead, there was long-distance gene flow between *S*. *purpurea* populations (S6 Table, Fig 7). This pattern supports the view that alpine plants are able to cover long distances through seed [46] or pollen dispersal [47].

Even if *S*. *purpurea* is capable of dispersing pollen over relatively long distances by wind, wind-based pollen dispersal is unlikely to be effective over such a long distance between the sampled populations. Anemochorous seed dispersal over large distances for *S*. *purpurea* is next to impossible [24]. Long-distance seed dispersal may be achieved with the assistance of native herbivores (e.g., the Tibetan antelope, *Pantholops hodgsonii*, also called chiru) and nomadic livestock (e.g., goats, sheep and yak). For instance, chiru females migrate a distance of around 300 km to the calving habitats in the northern QTP (e.g., Hoh Xil) in May and June of each year to give birth to their calves, and then migrate back to the wintering habitats in the southern QTP with their calves to reunite with males in August [48], just when *S*. *purpurea* is at the fruiting stage. *Stipa spp*. are the main fodder grasses of the chiru; their seeds easily attach to the body of chiru and may stay in the fur for hours or even days, resulting in long-distance zoochory [49]. Even if large proportions of the seeds are regularly damaged by the grazing herbivores, they might survive the digestive tract and still germinate [49]. Sufficient seed dispersal could occur to establish mammals an important gene flow mechanism over the long term.

### Ecological implications

In general, high levels of genetic diversity and unrestricted gene flow within a species are crucial for adaptation, especially when facing unpredictable local environmental changes [50]. The ability of a species to survive changes at their current location depends on their adaptive potential. Gene flow and selection are usually considered the main forces that affect the process of local adaptation. Our findings suggest that populations which are spread across a climatic gradient have the capacity to exchange genes by zoochorous seed dispersal. Such long-distance dispersal events, along with outcrossing, ensure population connectivity and large effective population sizes and reduce the effects of genetic drift, thus maintaining and transferring adaptive variation among populations in response to continuous climate change [16].

At the same time, population structure analysis and the morphological variation investigated here show that adaptive divergence between populations is associated with climatic factors. This finding is consistent with previous studies in perennial herb species, which found that patterns of local adaptation appear to be more common over large scales but rarer at small spatial scales [50]. It also suggests that high gene flow has not prevented the adaptive divergence of some populations of *S*. *purpurea* along environmental gradients. In particular, morphological variation analyses revealed that temperature and precipitation are likely to act as major drivers of selective responses in this alpine plant. This finding is consistent with previous studies that have demonstrated the prominent role of temperature and precipitation in the general adaptation of plants [51]. Such information is highly useful in modelling future vegetation dynamics under climate change. In addition, future studies will investigate the evolutionary and functional relevance of temperature and precipitation in alpine plants under experimental conditions.

## Supporting Information

S1 Fig AUC values of the 19 climate variables for *Stipa purpurea*. (TIF)

S2 Fig Principal-Coordinate-Analysis (PCoA) of the 19 climate variables. (TIF)

S3 Fig Principal-Coordinate-Analysis (PCoA) of the 14 morphological traits. (TIF)

S4 Fig Scatter plots of ΔK. The ΔK is based on the rate of change of ln P (X/K) between successive K values.
(TIF)

S1 Table Geographical characteristics and climatic variables of 20 populations of *Stipa purpurea*. (DOCX)

S2 Table SSR primers used and *F*-statistic for each marker. *Nm* = (1/4)(1-*Fst*)/*Fst*.
(DOCX)

S3 Table Descriptive statistics for 14 morphological traits of *S*. *purpurea*. *N*_*vshoot*_, the number of vegetative shoots; *L*_*vshoot*_, the height of vegetative shoots; *M*_*vshoot*_, the weight of vegetative shoots; *N*_*rshoot*_, the number of reproductive shoots; *L*_*spike*_, the height of the longest spike; *M*_*rshoot*_, the weight of reproductive shoots; *N*_*seed*_, the number of seeds; *M*_*100seed*_, the weight of 100 seeds; *N*_*root*_, the number of roots; *L*_*root*_, the length of roots; *M*_*root*_, the total weight of roots; *M*_*total*_, the total biomass; *RVR*, reproductive biomass/vegetative biomass; *RSR*, underground biomass/aboveground biomass.
(DOCX)

S4 Table Estimates of slope (*b*, allometric exponent) and intercept (*a*, allometric coefficient) for *logR-logV* regressions within populations of *S*. *purpurea*. Bold-faced slopes indicate a significant deviation from 1.
(DOCX)

S5 Table Effective population size of *S*. *purpurea* estimated by MIGRATE. *Ne* = *ϑ*/*μ* (*μ* = 10^−3^).
(DOCX)

S6 Table Estimates of bidirectional long-term gene flow (*M* = *m*/μ) between genetic regions using MIGRATE. The migration rates are from the populations in the vertical row into the populations in the horizontal column, 95% confidence interval is shown in brackets.
(DOCX)

S7 Table Estimates of bidirectional contemporary gene flow (*m*) between genetic regions using BAYESASS. (DOCX)

## References

[1] Davis MB, Shaw RG. Range shifts and adaptive responses to Quaternary climate change. Science. 2001 4; 292(5517): 673–679. 10.1126/science.292.5517.673 11326089

[2] Davis MB, Shaw RG, Etterson JR. Evolutionary responses to changing climate. Ecology. 2005 7; 86(7): 1704–1714. 10.1890/03-0788

[3] Gonzalo-Turpin H, Hazard L. Local adaptation occurs along altitudinal gradient despite the existence of gene flow in the alpine plant species *Festuca eskia*. J Ecol. 2009 4; 97(4): 742–751. 10.1111/j.1365-2745.2009.01509.x

[4] Franks SJ, Weber JJ, Aitken SN. Evolutionary and plastic responses to climate change in terrestrial plant populations. Evol Appl. 2014 1; 7(1): 123–139. 10.1111/eva.12112 24454552 PMC3894902

[5] Lind MI, Johansson F. Testing the role of phenotypic plasticity for local adaptation: growth and development in time-constrained *Rana temporaria* populations. J. Evol Biol. 2011 9; 24 (12): 2696–2704. 10.1111/j.1420-9101.2011.02393.x 21954876

[6] Johansson F, Veldhoen N, Lind MI, Helbing CC. Phenotypic plasticity in the hepatic transcriptome of the European common frog (*Rana temporaria*): the interplay between environmental induction and geographical lineage on developmental response. Mol Ecol 2013 11; 22(22): 5608–5623. 10.1111/mec.12497 24118477

[7] Wrange A, André C, Lundh T, Lind U, Blomberg A, Jonsson PJ, et al Importance of plasticity and local adaptation for coping with changing salinity in coastal areas: a test case with barnacles in the Baltic Sea. BMC Evol Biol. 2014 12; 14:156 10.1186/1471-2148-14-156 25038588 PMC4223505

[8] Hadfield JD. The spatial scale of local adaptation in a stochastic environment. Ecol Lett, 2016 5; 19(7): 780–788. 10.1111/ele.12614 27188689

[9] Parmesan C. Ecological and evolutionary responses to recent climate change. Annu Rev Ecol Evol Syst. 2006 12; 37(01): 637–669. 10.1146/annurev.ecolsys.37.091305.110100

[10] Byars SG, Papst W, Hoffmann AA. Local adaptation and cogradient selection in the alpine plant, *Poa hiemata*, along a narrow altitudinal gradient. Evolution. 2007 12; 61(12): 2925–2941. 10.1111/j.1558-5646.2007.00248.x 17924954

[11] McKay JK, Latta RG. Adaptive population divergence: markers, QTL and traits. Trends Ecol Evol. 2002 6; 17(02): 285–291. 10.1016/S0169-5347(02)02478-3

[12] Sexton JP, Hangartner SB, Hoffmann AA. Genetic isolation by environment or distance: which pattern of gene flow is most common? Evolution. 2014 1; 68(01): 1–15. 10.1111/evo.12258 24111567

[13] Blanquart F, Kaltz O, Nuismer SL, Gandon S. A practical guide to measuring local adaptation. Ecol Lett. 2013 7; 16(09): 1195–1205. 10.1111/ele.12150 23848550

[14] Byars SG, Parsons Y, Hoffmann AA. Effect of altitude on the genetic structure of an Alpine grass, *Poa hiemata*. Ann Bot. 2009 4; 103(06): 885–899. 10.1093/aob/mcp018 19208670 PMC2707893

[15] Sexton JP, Strauss SY, Rice KJ. Gene flow increases fitness at the warm edge of a species’ range. Proc Natl Acad Sci USA. 2011 7; 108(28):11704–11709. 10.1073/pnas.1100404108 21709253 PMC3136252

[16] Kremer A, Ronce O, Robledo-Arnuncio JJ, Guillaume F, Bohrer G, Nathan R, et al Long-distance gene flow and adaptation of forest trees to rapid climate change. Ecol Lett. 2012 2; 15(04): 378–392. 10.1111/j.1461-0248.2012.01746.x 22372546 PMC3490371

[17] Weiner J, Campbell LG, Pino J, Echarte L. The allometry of reproduction within plant populations. J Ecol. 2009 9; 97(06): 1220–1233. 10.1111/j.1365-2745.2009.01559.x

[18] Vasseur F, Violle C, Enquist BJ, Granier C, Vile D. A common genetic basis to the origin of the leaf economics spectrum and metabolic scaling allometry. Ecol Lett. 2012 7; 15(10): 1149–1157. 10.1111/j.1461-0248.2012.01839.x 22856883

[19] Weppler T, Stoll P, Stӧcklin J. The relative importance of sexual and clonal reproduction for population growth in the long-lived alpine plant *Geum reptans*. J Ecol. 2006 5; 94(04): 869–879. 10.1111/j.1365-2745.2006.01134.x

[20] Guo H, Weiner J, Mazer SJ, Zhao Z, Du G, Li B. Reproductive allometry in Pedicularis species changes with elevation. J Ecol. 2012 3; 100(02): 452–458. 10.1111/j.1365-2745.2011.01884.x

[21] Myers N, Russell M, Cristina M, Gustavo DF, Jennifer K. Biodiversity hotspots for conservation priorities. Nature. 2000 3; 403(6772): 853–858. 10.1038/35002501 10706275

[22] Liu X, Chen B. Climatic warming in the Tibetan Plateau during recent decades. Int J Climatol. 2000 11; 20(14): 1729–1742. 10.1002/1097-0088

[23] Yue PP, Lu XF, Ye RR, Zhang CX, Yang SB, Zhou YB, et al Distribution of *Stipa purpurea* steppe in the Northeastern Qinghai-Xizang Plateau (China). Russ J Ecol. 2011 1; 42(01): 50–56. 10.1134/S1067413611010140

[24] Liu WS, Dong M, Song ZP, Wei W. Genetic diversity pattern of *Stipa purpurea* populations in the hinterland of Qinghai-Tibet Plateau. Ann Appl Biol. 2009 1; 154(01): 57–65. 10.1111/j.1744-7348.2008.00274.x

[25] Zhang YL, Li BY, Zheng D. A discussion on the boundary and area of the Tibetan Plateau in China (in Chinese with English abstract). Geogr Res. 2002 1; 21(01): 1–10.

[26] Phillips SJ, Anderson RP, Schapire RE. Maximum entropy modeling of species geographic distributions. Ecol Modell. 2006 1; 190(3–4): 231–259. 10.1016/j.ecolmodel.2005.03.026

[27] Klinkhamer PGL, Meelis E, de Jong TJ, Weiner J. On the analysis of size-dependent reproductive output in plants. Funct Ecol. 1992 3; 6(03): 308–316. 10.2307/2389522

[28] Falster DS, Warton DI, Wright IJ. User’s guide to SMATR: Standardised major axis tests and routines. Version 2.0, http://www.bio.mq.edu.au/ecology/SMATR. 2006.

[29] Liu WS, Liao H, Zhou Y, Zhao Y, Song ZP. Microsatellite primers in *Stipa purpurea* (Poaceae), a dominant species of the steppe on the Qinghai-Tibetan Plateau. Am J Bot. 2011 2; 98(06): 150–151. 10.3732/ajb.1000444 21613069

[30] Van Oosterhout C, Hutchinson WF, Wills DPM, Shipley P. Microchecker: software for identifying and correcting genotyping errors in microsatellite data. Mol Ecol Notes. 2004 7; 4(03): 535–538. 10.1111/j.1471-8286.2004.00684.x

[31] Peakall R, Smouse PE. GenAlEx: genetic analysis in Excel Canberra: Australian National University 2001.

[32] Evanno G, Regnaut S, Goudet J. Detecting the number of clusters of individuals using the software STRUCTURE: a simulation study. Mol Ecol. 2005 5; 14(08): 2611–2620. 10.1111/j.1365-294X.2005.02553.x 15969739

[33] Dieringer D, Schlötterer C. Microsatellite analyser (MSA): a platform independent analysis tool for large microsatellite data sets. Mol Ecol Notes. 2003 1; 3(01): 167–169. 10.1046/j.1471-8286.2003.00351.x

[34] Beerli P. Migrate version 3.0-a maximum likelihood and Bayesian estimator of gene flow using the coalescent. Available at http://popgen.scs.edu/migrate.html. 2008.

[35] Wilson GA, Rannala B. Bayesian inference of recent migration rates using multilocus genotypes. Genetics. 2003 3; 163(3): 1177–1191. 12663554 10.1093/genetics/163.3.1177 PMC1462502

[36] Faubet P, Waples R, Gaggiotti O. Evaluating the performance of a multi−locus Bayesian method for the estimation of migration rates. Mol Ecol. 2007 3; 16(6): 1149–1166. 10.1111/j.1365-294X.2007.03218.x 17391403

[37] Ronnenberg K, Hensen I, Wesche K. Contrasting effects of precipitation and fertilization on seed viability and production of *Stipa krylovii* in Mongolia. Basic Appl Ecol. 2011 3; 12(02): 141–151. 10.1016/j.baae.2010.12.002

[38] Hendrix SD, Nielsen E, Nielsen T, Schutt M. Are seedlings from small seeds always inferior to seedlings from large seeds—Effects of seed biomass on seedling growth in Pastinaca-Sativa L. New Phytol. 1991 10; 119(02): 299–305. 10.1111/j.1469-8137.1991.tb01034.x 33874142

[39] Luo WT, Jiang Y, Lu XT, Wang X, Li MH, Bai E, et al Patterns of Plant Biomass Allocation in Temperate Grasslands across a 2500-km Transect in Northern China. PloS One. 2013 8; 8(8): e71749 10.1371/journal.pone.0071749 23977135 PMC3748100

[40] Durka W, Nosso C, Welk E, Ruprecht E, Wagner V, Wesche K, et al Extreme genetic depauperation and differentiation of both populations and species in Eurasian feather grasses (*Stipa*). Plant Syst Evol. 2013 1; 299(01): 259–269. 10.1007/s00606-012-0719-0

[41] Hamasha HR, Schmidt-Lebuhn AN, Durka W, Schleuning M, Hensen I. Bioclimatic regions influence genetic structure of four Jordanian *Stipa* species. Plant Biol. 2013 9; 15(05): 882–891. 10.1111/j.1438-8677.2012.00689.x 23369254

[42] Shen DF, Bo WH, Xu F, Wu RL. Genetic diversity and population structure of the Tibetan poplar (*Populus szechuanica* var. *tibetica*) along an altitude gradient. BMC Genet. 2014 6; 15(Suppl 1): S11 10.1186/1471-2156-15-S1-S11 25079034 PMC4118629

[43] Wang Z, Kang M, Liu H, Gao J, Zhang Z, Li Y, et al High-level genetic diversity and complex population structure of Siberian apricot (*Prunus sibirica* L.) in China as revealed by nuclear SSR markers. PLoS One. 2014 2; 9(02): e87381 10.1371/journal.pone.0087381 24516551 PMC3917850

[44] Stoeckel S, Grange J, Fernández-Manjarres JF, Bilger I, Frascaria-Lacoste N, Mariette S. Heterozygote excess in a self-incompatible and partially clonal forest tree species *Prunus avium* L. Mol Ecol. 2006 7; 15(08): 2109–2118. 10.1111/j.1365-294X.2006.02926.x 16780428

[45] Kawecki TJ. Adaptation to marginal habitats. Annu Rev Ecol Evol Syst. 2008 10; 39(04): 321–342. 10.1146/annurev.ecolsys.38.091206.095622

[46] Alsos IG, Eidesen PB, Ehrich D, Skrede I, Westergaard K, Jacobsen GH, et al Frequent long distance colonization in the changing Arctic. Science. 2007 6; 316(5831): 1606–1609. 10.1126/science.1139178 17569861

[47] Garcia-Fernandez A, Segarra-Moragues JG, Widmer A, Escudero A, Iriondo JM. Unravelling genetics at the top: mountain islands or isolated belts? Ann Bot. 2012 9; 110(06): 1221–1232. 10.1093/aob/mcs195 23002271 PMC3478054

[48] Zhang F, Jiang Z, Xu A, Zeng Y, Li C. Recent Geological Events and Intrinsic Behavior Influence the Population Genetic Structure of the Chiru and Tibetan Gazelle on the Tibetan Plateau. PLoS One. 2013 4; 8(4): e60712 10.1371/journal.pone.0060712 23637761 PMC3634780

[49] Bläß C, Ronnenberg K, Tackenbergb O, Hensena I, Weschec K. The relative importance of different seed dispersal modes in dry Mongolian rangelands. J Arid Environ. 2010 8; 74(08): 991–997. 10.1016/j.jaridenv.2009.12.002

[50] Matter P, Kettle CJ, Ghazoul J, Pluess AR. Extensive contemporary pollen-mediated gene flow in two herb species, *Ranunculus bulbosus* and *Trifolium montanum*, along an altitudinal gradient in a meadow landscape. Ann Bot. 2013 4; 111(04): 611–621. 10.1093/aob/mct021 23408831 PMC3605955

[51] Manel S, Gugerli F, Thuiller W, Alvarez N, Legendre P, Holderegger R, et al Broad-scale adaptive genetic variation in alpine plants is driven by temperature and precipitation. Mol Ecol. 2012 8; 21(15): 3729–3738. 10.1111/j.1365-294X.2012.05656.x 22680783 PMC4003392

