# Supplementary figures and images for: Morphological and Genetic Variation along a North-to-South Transect in Stipa purpurea, a Dominant Grass on the Qinghai-Tibetan Plateau: Implications for Response to Climate Change
Source: PLoS One. 2016 Aug 31;11(8):e0161972. doi: 10.1371/journal.pone.0161972 (PMC5006974; doi:10.1371/journal.pone.0161972)

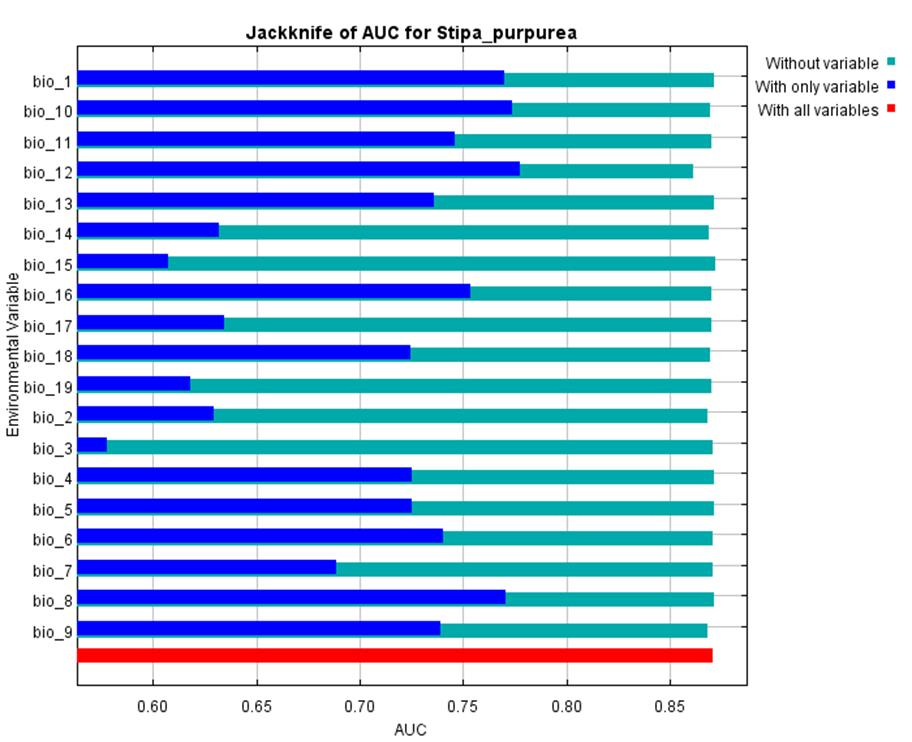

Supplement: S1 Fig — (TIF) [file pone.0161972.s001.tif]

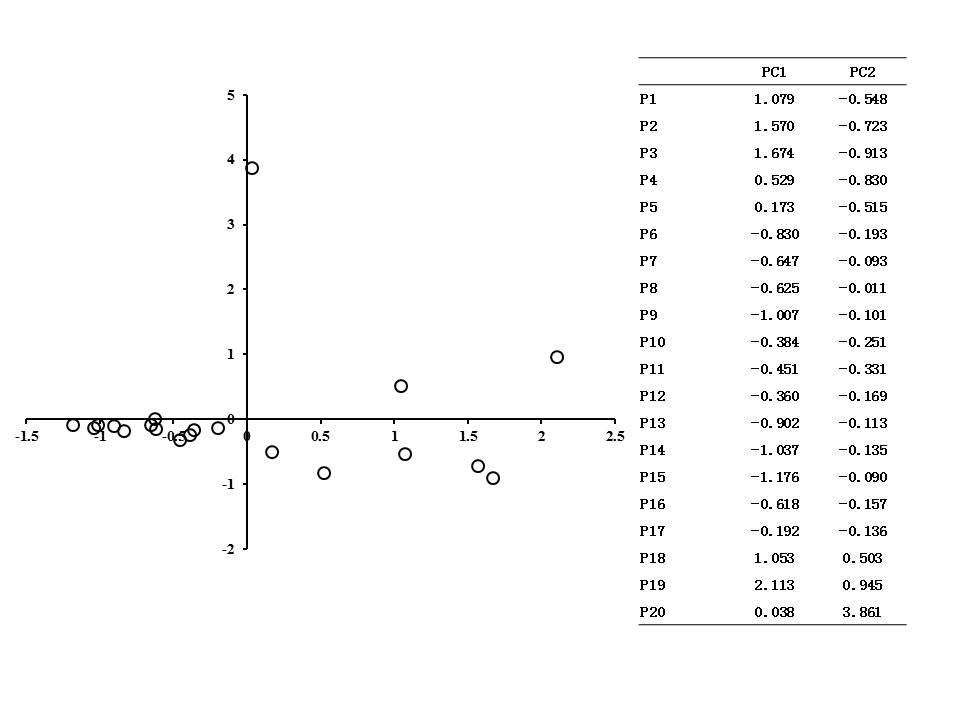

Supplement: S2 Fig — (TIF) [file pone.0161972.s002.tif]

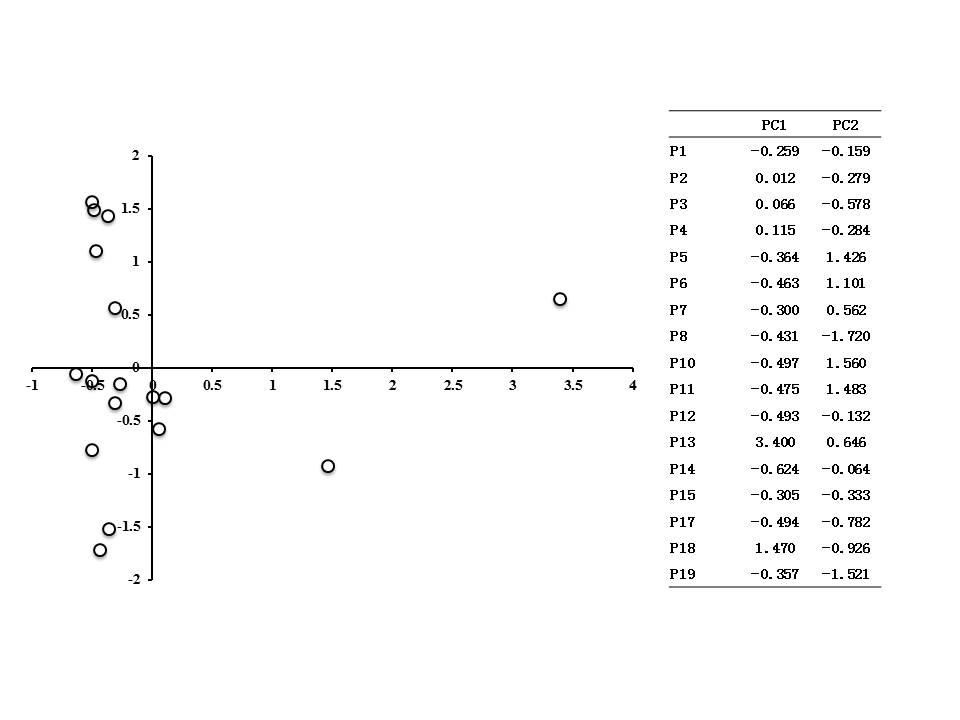

Supplement: S3 Fig — (TIF) [file pone.0161972.s003.tif]

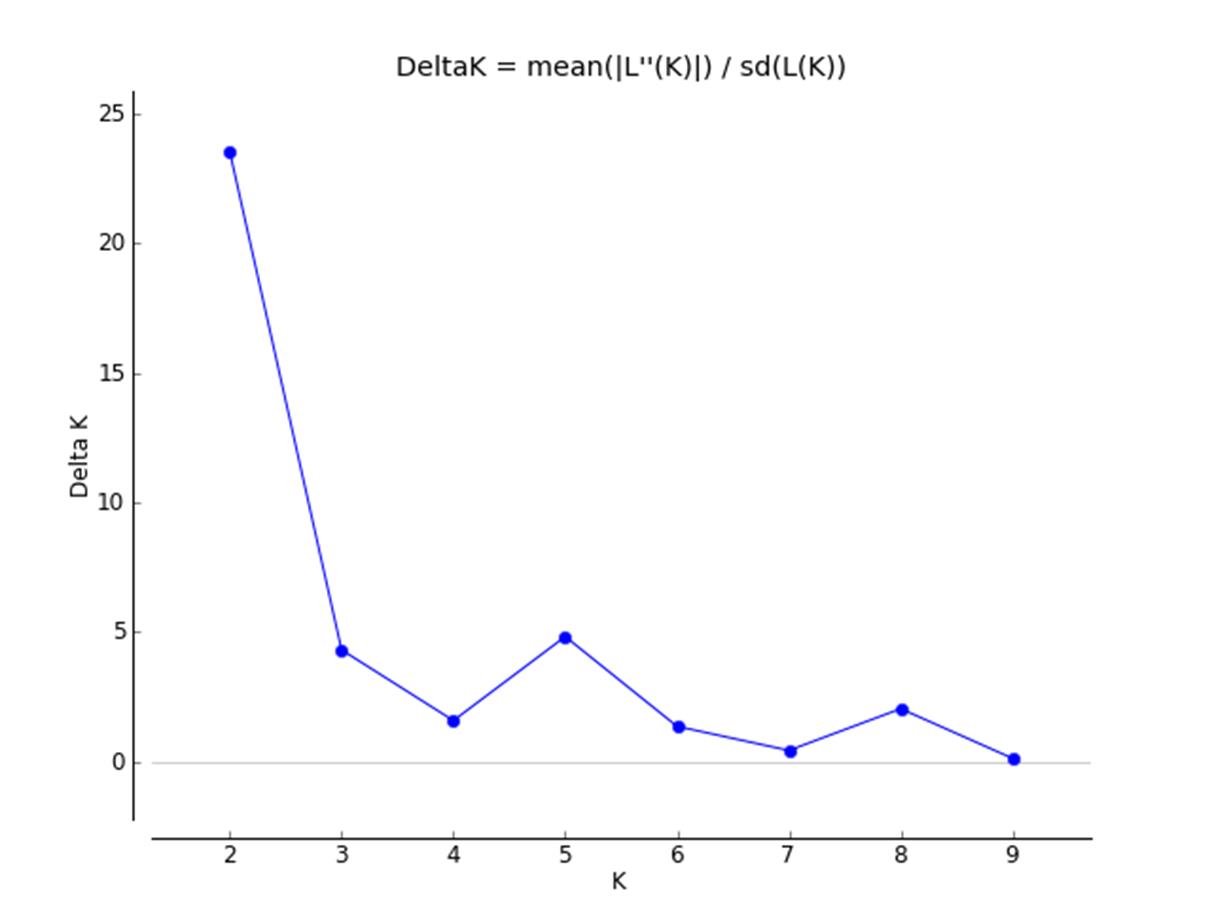

Supplement: S4 Fig — The ΔK is based on the rate of change of ln P (X/K) between successive K values. (TIF) [file pone.0161972.s004.tif]
