# Supplementary material for: Morphological and Genetic Variation along a North-to-South Transect in Stipa purpurea, a Dominant Grass on the Qinghai-Tibetan Plateau: Implications for Response to Climate Change
Source: PLoS One. 2016 Aug 31;11(8):e0161972. doi: 10.1371/journal.pone.0161972 (PMC5006974; doi:10.1371/journal.pone.0161972)
Supplement: S1 Table — (DOCX) [file pone.0161972.s005.docx]

**S1 Table Geographical characteristics and climatic variables of 20 populations of *Stipa purpurea***

| **Pop** | **Site** | **Longitude (E)** | **Latitude (N)** | **Altttude (m)** | **bio1** | **bio2** | **bio3** | **bio4** | **bio5** | **bio6** | **bio7** | **bio8** | **bio9** | **bio10** | **bio11** | **bio12** | **bio13** | **bio14** | **bio15** | **bio16** | **bio17** | **bio18** | **bio19** |
| --- | --- | --- | --- | --- | --- | --- | --- | --- | --- | --- | --- | --- | --- | --- | --- | --- | --- | --- | --- | --- | --- | --- | --- |
| 1 | Qinghaihu 1 | 100.69 | 37.08 | 3498 | -0.95 | 12.86 | 35.23 | 849.04 | 15.30 | -21.20 | 36.50 | 8.90 | -10.75 | 8.90 | -11.97 | 404 | 93 | 1 | 105.31 | 252 | 5 | 252 | 5 |
| 2 | Qinghaihu 2 | 100.02 | 37.27 | 3245 | 0.35 | 13.18 | 35.42 | 871.42 | 17.00 | -20.20 | 37.20 | 10.40 | -10.97 | 10.40 | -10.97 | 349 | 82 | 1 | 108.58 | 224 | 4 | 224 | 4 |
| 3 | Qinghaihu 3 | 99.77 | 37.18 | 3206 | 0.64 | 13.66 | 35.48 | 900.43 | 18.00 | -20.50 | 38.50 | 11.07 | -9.92 | 11.07 | -11.02 | 301 | 70 | 1 | 108.07 | 193 | 4 | 193 | 4 |
| 4 | Qinghaihu 4 | 98.87 | 37.11 | 3768 | -1.75 | 13.81 | 35.50 | 918.09 | 16.30 | -22.60 | 38.90 | 9.07 | -12.42 | 9.07 | -13.38 | 248 | 53 | 1 | 102.90 | 156 | 5 | 156 | 6 |
| 5 | Xidatan | 94.32 | 35.75 | 4180 | -2.80 | 14.29 | 37.03 | 903.18 | 15.30 | -23.30 | 38.60 | 8.02 | -13.12 | 8.02 | -14.05 | 164 | 42 | 1 | 112.16 | 108 | 3 | 108 | 3 |
| 6 | Budongquan | 93.91 | 35.52 | 4615 | -5.03 | 13.99 | 37.51 | 864.11 | 12.50 | -24.80 | 37.30 | 5.37 | -14.70 | 5.37 | -15.70 | 222 | 58 | 1 | 114.61 | 148 | 3 | 148 | 4 |
| 7 | Suonandajie | 93.75 | 35.52 | 4561 | -4.74 | 14.01 | 37.76 | 860.77 | 12.70 | -24.40 | 37.10 | 5.62 | -14.35 | 5.62 | -15.37 | 220 | 58 | 1 | 115.58 | 148 | 3 | 148 | 4 |
| 8 | Erdaodaogou | 92.93 | 34.81 | 4637 | -4.56 | 14.36 | 39.23 | 843.24 | 12.60 | -24.00 | 36.60 | 5.57 | -13.88 | 5.57 | -14.93 | 279 | 78 | 1 | 120.17 | 192 | 3 | 192 | 4 |
| 9 | Tuotuohe | 92.78 | 34.61 | 4770 | -5.09 | 14.63 | 39.63 | 846.98 | 12.10 | -24.80 | 36.90 | 5.10 | -14.45 | 5.10 | -15.45 | 284 | 82 | 1 | 122.13 | 198 | 3 | 198 | 4 |
| 10 | Buderka | 92.67 | 34.39 | 4561 | -3.76 | 15.01 | 39.92 | 862.38 | 13.70 | -23.90 | 37.60 | 6.57 | -13.42 | 6.57 | -14.35 | 284 | 84 | 0 | 123.85 | 200 | 2 | 200 | 4 |
| 11 | Tuotuohe | 92.48 | 34.27 | 4567 | -3.68 | 15.34 | 40.37 | 869.47 | 13.90 | -24.10 | 38.00 | 6.73 | -13.42 | 6.73 | -14.35 | 281 | 85 | 0 | 125.54 | 200 | 2 | 200 | 4 |
| 12 | Tangganmu | 92.28 | 33.79 | 4616 | -3.50 | 15.22 | 40.15 | 862.11 | 14.00 | -23.90 | 37.90 | 6.83 | -13.15 | 6.83 | -14.10 | 309 | 90 | 0 | 122.91 | 217 | 4 | 217 | 6 |
| 13 | Yanshiping | 92.08 | 33.71 | 4795 | -4.45 | 15.08 | 40.09 | 857.28 | 12.90 | -24.70 | 37.60 | 5.83 | -14.00 | 5.83 | -14.97 | 305 | 89 | 1 | 123.17 | 215 | 5 | 215 | 6 |
| 14 | Tanggulabei | 91.87 | 33.14 | 4936 | -4.75 | 14.88 | 39.77 | 845.45 | 12.40 | -25.00 | 37.40 | 5.38 | -14.15 | 5.38 | -15.17 | 327 | 92 | 1 | 121.07 | 227 | 5 | 227 | 6 |
| 15 | Tanggula | 91.89 | 32.75 | 5055 | -5.04 | 14.79 | 39.87 | 835.44 | 11.80 | -25.30 | 37.10 | 4.97 | -14.32 | 4.97 | -15.33 | 343 | 94 | 1 | 120.33 | 237 | 5 | 237 | 6 |
| 16 | Tanggulanan | 91.74 | 32.40 | 4861 | -3.67 | 14.88 | 39.90 | 832.34 | 13.20 | -24.10 | 37.30 | 6.25 | -12.97 | 6.25 | -14.00 | 355 | 95 | 2 | 119.54 | 244 | 6 | 244 | 6 |
| 17 | Anduo | 91.68 | 32.10 | 4750 | -2.81 | 14.93 | 40.12 | 828.92 | 13.90 | -23.30 | 37.20 | 7.03 | -12.12 | 7.03 | -13.13 | 366 | 97 | 2 | 118.65 | 250 | 6 | 250 | 6 |
| 18 | Guluzhen | 91.66 | 30.97 | 4735 | -1.18 | 14.94 | 41.50 | 780.58 | 14.70 | -21.30 | 36.00 | 8.08 | -9.32 | 8.08 | -10.92 | 349 | 94 | 1 | 124.18 | 246 | 4 | 246 | 4 |
| 19 | Dangxiong | 91.46 | 30.57 | 4628 | 0.25 | 14.87 | 42.36 | 751.14 | 15.70 | -19.40 | 35.10 | 9.17 | -7.58 | 9.17 | -9.13 | 326 | 91 | 0 | 130.62 | 238 | 2 | 238 | 2 |
| 20 | Everest 5000 | 86.84 | 28.17 | 5628 | -4.96 | 14.27 | 46.17 | 624.77 | 8.50 | -22.40 | 30.90 | 2.17 | -11.37 | 2.68 | -12.50 | 300 | 100 | 1 | 134.68 | 222 | 10 | 213 | 13 |
