# Supplementary material for: Morphological and Genetic Variation along a North-to-South Transect in Stipa purpurea, a Dominant Grass on the Qinghai-Tibetan Plateau: Implications for Response to Climate Change
Source: PLoS One. 2016 Aug 31;11(8):e0161972. doi: 10.1371/journal.pone.0161972 (PMC5006974; doi:10.1371/journal.pone.0161972)
Supplement: S2 Table — Nm = (1/4)(1-Fst)/Fst. (DOCX) [file pone.0161972.s006.docx]

**S2 Table SSR primers used and *F*-statistic for each marker**

| primer | motif | *Fis* | *Fit* | *Fst* | *Nm*^a^ |
| --- | --- | --- | --- | --- | --- |
| **SP18** | (CAT)_2_(CCT)_3_(CAT)_3_(CCT)_2_(CAT)_3_CA | -0.021 | 0.068 | 0.088 | 2.595 |
| **SP90** | (GAT)_11_ | -0.124 | 0.055 | 0.159 | 1.318 |
| **SP396** | (CT)_8_ | -0.075 | 0.096 | 0.158 | 1.328 |
| **SP152** | (TC)_11_ | -0.152 | 0.131 | 0.246 | 0.768 |
| **SP182** | (GA)_13_ | -0.041 | 0.127 | 0.161 | 1.304 |
| **SP419** | AGG(AAG)_7_ | -0.050 | 0.255 | 0.291 | 0.609 |
| **SP185** | AAG(AGC)_5_AGG | -0.035 | 0.125 | 0.154 | 1.369 |
| **SP199** | (TGA)_8_TGG | -0.327 | -0.133 | 0.146 | 1.462 |
| **SP441** | (GA)_11_GCGAGC(GA)_7_GGA | -0.095 | 0.226 | 0.293 | 0.604 |
| **SP202** | (CT)_14_ | -0.063 | 0.146 | 0.196 | 1.023 |
| **SP207** | (TC)_9_ | -0.098 | 0.211 | 0.281 | 0.640 |
| **Mean** |  | -0.098 | 0.119 | 0.198 | 1.184 |
| **SE** |  | 0.026 | 0.032 | 0.021 | 0.174 |

^a^*Nm*= (1/4)(1-*Fst*)/*Fst*
