# Supplementary material for: Morphological and Genetic Variation along a North-to-South Transect in Stipa purpurea, a Dominant Grass on the Qinghai-Tibetan Plateau: Implications for Response to Climate Change
Source: PLoS One. 2016 Aug 31;11(8):e0161972. doi: 10.1371/journal.pone.0161972 (PMC5006974; doi:10.1371/journal.pone.0161972)
Supplement: S3 Table — Nvshoot, the number of vegetative shoots; Lvshoot, the height of vegetative shoots; Mvshoot, the weight of vegetative shoots; Nrshoot, the number of reproductive shoots; Lspike, the height of the longest spike; Mrshoot, the weight of reproductive shoots; Nseed, the number of seeds; M100seed, the weight of 100 seeds; Nroot, the number of roots; Lroot, the length of roots; Mroot, the total weight of roots; Mtotal, the total biomass; RVR, reproductive biomass/vegetative biomass; RSR, underground biomass/aboveground biomass. (DOCX) [file pone.0161972.s007.docx]

**S3 Table Descriptive statistics for 14 morphological traits of *S. purpurea***

| **Pop** | | ***N_vshoot_*** | ***L_vshoot_***  **(mm)** | ***M_vshoot_***  **(g)** | ***N_rshoot_*** | ***L_spike_***  **(mm)** | ***M_rshoot_***  **(g)** | ***N_seed_*** | ***M_100seeds_***  **(g)** | ***N_root_*** | ***L_root_***  **(mm)** | ***M_root_***  **(g)** | ***M_total_***  **(g)** | ***RVR*** | ***RSR*** |
| --- | --- | --- | --- | --- | --- | --- | --- | --- | --- | --- | --- | --- | --- | --- | --- |
| **P1** | Mean | 64.25 | 12.21 | 1.1 | 5.45 | 22.35 | 0.81 | 16.3 | 1.53 | 75.55 | 5.46 | 1.01 | 2.92 | 0.29 | 0.55 |
|  | SE | 11.26 | 0.31 | 0.1 | 0.91 | 0.81 | 0.05 | 0.56 | 1.79 | 6.64 | 0.28 | 0.07 | 0.18 | 0.02 | 0.03 |
|  | min | 12 | 9.35 | 0.47 | 1 | 17.4 | 0.5 | 13 | 1.31 | 15 | 2.56 | 0.59 | 1.91 | 0.15 | 0.31 |
|  | max | 204 | 14.61 | 2 | 17 | 27.56 | 1.33 | 21 | 1.62 | 128 | 8.07 | 1.82 | 4.49 | 0.43 | 0.77 |
| **P2** | Mean | 89.6 | 10.29 | 1.55 | 8 | 19.73 | 1.17 | 14 | 1.36 | 101.8 | 6.36 | 1.5 | 4.22 | 0.28 | 0.56 |
|  | SE | 13.01 | 0.57 | 0.15 | 1.56 | 0.79 | 0.14 | 0.77 | 1.30 | 10.45 | 0.2 | 0.16 | 0.34 | 0.02 | 0.05 |
|  | min | 67 | 7.38 | 1.18 | 3 | 14.86 | 0.74 | 10 | 1.40 | 53 | 5.2 | 0.74 | 2.8 | 0.18 | 0.34 |
|  | max | 165 | 13.23 | 2.39 | 18 | 19.74 | 1.94 | 17 | 1.59 | 173 | 7.51 | 2.3 | 4.89 | 0.36 | 0.89 |
| **P3** | Mean | 89.6 | 8.15 | 1.23 | 19 | 20.98 | 1.37 | 13.8 | 1.96 | 99.2 | 6.25 | 1.08 | 3.69 | 0.37 | 0.42 |
|  | SE | 12.34 | 0.42 | 0.12 | 2.8 | 0.54 | 0.1 | 1.07 | 1.87 | 17.02 | 0.25 | 0.08 | 0.22 | 0.02 | 0.03 |
|  | min | 16 | 6.42 | 0.76 | 4 | 18.63 | 0.79 | 9 | 1.67 | 43 | 4.46 | 0.7 | 2.88 | 0.27 | 0.31 |
|  | max | 147 | 10.25 | 1.58 | 30 | 23.5 | 1.87 | 19 | 1.95 | 230 | 7.28 | 1.54 | 5.28 | 0.44 | 0.57 |
| **P4** | Mean | 104.7 | 8.9 | 1.38 | 8.7 | 17.68 | 1.08 | 14.6 | 1.99 | 150 | 6.15 | 1.31 | 3.76 | 0.29 | 0.52 |
|  | SE | 14.85 | 0.41 | 0.12 | 2.33 | 0.47 | 0.15 | 0.86 | 2.33 | 19.42 | 0.27 | 0.19 | 0.32 | 0.03 | 0.06 |
|  | min | 56 | 7.24 | 0.88 | 2 | 15.63 | 0.51 | 10 | 2.10 | 68 | 4.76 | 0.29 | 2.28 | 0.16 | 0.15 |
|  | max | 198 | 10.87 | 1.97 | 22 | 20.17 | 2.06 | 18 | 1.89 | 245 | 7.53 | 2.17 | 5.24 | 0.45 | 0.77 |
| **P5** | Mean | 68.7 | 9.44 | 0.77 | 2.9 | 19.19 | 0.2 | 13.3 | 2.86 | 124.4 | 13.67 | 1.28 | 2.24 | 0.1 | 1.44 |
|  | SE | 7.05 | 0.95 | 0.1 | 0.57 | 2.2 | 0.03 | 1.18 | 4.24 | 17.39 | 0.79 | 0.22 | 0.29 | 0.02 | 0.26 |
|  | min | 38 | 4.98 | 0.27 | 1 | 7.1 | 0.04 | 8 | 2.38 | 62 | 9.83 | 0.27 | 1.24 | 0.01 | 0.28 |
|  | max | 97 | 15.74 | 1.27 | 7 | 31.9 | 0.31 | 20 | 3.80 | 212 | 18.14 | 2.7 | 4.28 | 0.2 | 3.11 |
| **P6** | Mean | 63.4 | 10.37 | 1.18 | 8.4 | 16.24 | 0.88 | 6.6 | 2.27 | 88.3 | 13.14 | 1.44 | 3.5 | 0.24 | 0.78 |
|  | SE | 5.6 | 0.6 | 0.12 | 1.39 | 0.68 | 0.13 | 0.45 | 2.22 | 12.01 | 0.96 | 0.11 | 0.28 | 0.02 | 0.09 |
|  | min | 39 | 7.23 | 0.8 | 3 | 13.22 | 0.3 | 5 | 2.00 | 49 | 8.38 | 1.01 | 2.45 | 0.12 | 0.34 |
|  | max | 102 | 13.09 | 1.94 | 17 | 19.7 | 1.49 | 9 | 2.44 | 172 | 16.97 | 2.06 | 4.53 | 0.32 | 1.22 |
| **P7** | Mean | 75.7 | 9.92 | 1.21 | 11.7 | 15.42 | 1.06 | 6.5 | 2.15 | 108.6 | 10.59 | 1.32 | 3.59 | 0.3 | 0.6 |
|  | SE | 17.9 | 0.41 | 0.13 | 2.84 | 0.43 | 0.1 | 0.31 | 3.23 | 15.56 | 0.63 | 0.09 | 0.27 | 0.02 | 0.04 |
|  | min | 22 | 8.17 | 0.72 | 4 | 13.68 | 0.79 | 5 | 2.00 | 63 | 8.67 | 0.78 | 2.44 | 0.23 | 0.47 |
|  | max | 195 | 10.71 | 2.05 | 31 | 16.67 | 1.73 | 8 | 2.38 | 238 | 14.98 | 1.64 | 5.11 | 0.39 | 0.8 |
| **P8** | Mean | 27.43 | 4.34 | 0.1 | 4.43 | 9.26 | 0.06 | 6.57 | 1.07 | 30 | 4.66 | 0.08 | 0.24 | 0.29 | 0.53 |
|  | SE | 6.06 | 0.22 | 0.03 | 1.23 | 0.97 | 0.01 | 0.37 | 2.70 | 4.01 | 0.38 | 0.04 | 0.05 | 0.06 | 0.16 |
|  | min | 14 | 3.5 | 0.04 | 1 | 6.43 | 0.02 | 5 | 1.00 | 22 | 3.36 | 0.02 | 0.13 | 0.07 | 0.08 |
|  | max | 50 | 4.95 | 0.23 | 9 | 14.25 | 0.11 | 8 | 1.13 | 45 | 6.52 | 0.3 | 0.54 | 0.47 | 1.25 |
| **P10** | Mean | 109 | 9.75 | 1.26 | 3 | 15.29 | 0.13 | 6.9 | 3.19 | 71.25 | 10.9 | 0.8 | 2.19 | 0.07 | 0.77 |
|  | SE | 13.78 | 0.64 | 0.29 | 0.33 | 0.89 | 0.02 | 0.38 | 5.26 | 9.42 | 0.95 | 0.09 | 0.34 | 0.01 | 0.16 |
|  | min | 57 | 7.16 | 0.19 | 2 | 12.5 | 0.03 | 5 | 3.00 | 47 | 8.47 | 0.54 | 0.85 | 0.01 | 0.32 |
|  | max | 167 | 12.8 | 2.31 | 4 | 18.73 | 0.19 | 9 | 3.22 | 125 | 15.94 | 1.27 | 3.27 | 0.14 | 1.74 |
| **P11** | Mean | 102.9 | 13.61 | 1.74 | 5 | 21.54 | 0.48 | 8.2 | 2.32 | 84.9 | 10.46 | 0.93 | 3.14 | 0.16 | 0.44 |
|  | SE | 16.68 | 0.67 | 0.32 | 1.08 | 1.18 | 0.1 | 0.51 | 1.96 | 10.77 | 0.8 | 0.15 | 0.52 | 0.03 | 0.05 |
|  | min | 50 | 10.93 | 0.71 | 1 | 15.2 | 0.18 | 7 | 2.14 | 34 | 6.74 | 0.35 | 1.46 | 0.06 | 0.29 |
|  | max | 196 | 16.67 | 4.04 | 11 | 28.18 | 1.09 | 11 | 2.36 | 130 | 14.84 | 1.99 | 6.79 | 0.32 | 0.79 |
| **P12** | Mean | 47.3 | 10.33 | 1.03 | 1.5 | 21.12 | 0.61 | 7.25 | 1.10 | 46.3 | 7.95 | 0.8 | 2.44 | 0.26 | 0.5 |
|  | SE | 4.43 | 0.19 | 0.08 | 0.84 | 0.5 | 0.05 | 0.35 | 11.43 | 4.69 | 0.28 | 0.05 | 0.11 | 0.01 | 0.07 |
|  | min | 34 | 8.34 | 0.5 | 1 | 17.6 | 0.45 | 7 | 0.86 | 20 | 5.78 | 0.57 | 1.52 | 0.19 | 0.31 |
|  | max | 73 | 13.91 | 1.56 | 2 | 24.7 | 0.9 | 8 | 1.50 | 73 | 10.03 | 1.01 | 2.97 | 0.3 | 0.57 |
| **P13** | Mean | 321.25 | 8.27 | 4.08 | 2.75 | 11.18 | 0.06 | 6.5 | 2.77 | 0.47 | 13.16 | 0.97 | 5.1 | 0.01 | 0.38 |
|  | SE | 125.33 | 0.36 | 1.97 | 0.75 | 1.69 | 0.02 | 0.22 | 9.09 | 0.21 | 1.05 | 0.33 | 0.33 | 0.04 | 11.09 |
|  | min | 184 | 7.2 | 1.8 | 1 | 8.4 | 10 | 6 | 2.00 | 228 | 10.66 | 496 | 0.58 | 0.02 | 4 |
|  | max | 697 | 8.76 | 10 | 4 | 16 | 114 | 8 | 4.00 | 1082 | 15.8 | 1941 | 1.99 | 0.18 | 48.16 |
| **P14** | Mean | 17.2 | 8.46 | 0.62 | 1.7 | 20.25 | 0.44 | 5.9 | 2.03 | 48.9 | 7 | 0.55 | 1.61 | 0.25 | 0.51 |
|  | SE | 2.76 | 0.47 | 0.06 | 0.26 | 1.76 | 0.08 | 0.23 | 4.35 | 7.4 | 0.4 | 0.09 | 0.21 | 0.03 | 0.04 |
|  | min | 4 | 6.35 | 0.41 | 1 | 14 | 0.04 | 5 | 2.00 | 15 | 4.72 | 0.17 | 0.62 | 0.06 | 0.38 |
|  | max | 33 | 11 | 0.86 | 3 | 30.2 | 0.75 | 7 | 2.43 | 84 | 8.78 | 1.03 | 2.46 | 0.34 | 0.74 |
| **P15** | Mean | 36.7 | 8.38 | 1.01 | 1.7 | 16.74 | 0.81 | 5.9 | 1.53 | 118.1 | 7.04 | 1.08 | 2.9 | 0.28 | 0.6 |
|  | SE | 6.66 | 0.19 | 0.19 | 0.3 | 0.67 | 0.18 | 0.23 | 4.35 | 22.53 | 0.35 | 0.21 | 0.57 | 0.02 | 0.04 |
|  | min | 14 | 7.4 | 0.46 | 1 | 13.15 | 0.47 | 5 | 1.20 | 41 | 5.65 | 0.53 | 1.8 | 0.21 | 0.4 |
|  | max | 82 | 9.27 | 2.59 | 4 | 19.5 | 2.39 | 7 | 1.86 | 240 | 8.61 | 2.83 | 7.81 | 0.35 | 0.78 |
| **P17** | Mean | 22.2 | 7.56 | 0.22 | 2.7 | 21.44 | 0.14 | 12.6 | 2.14 | 31 | 3.17 | 0.21 | 0.57 | 0.26 | 0.71 |
|  | SE | 3.83 | 0.35 | 0.03 | 0.97 | 2.16 | 0.03 | 0.99 | 10.10 | 3.78 | 0.22 | 0.06 | 0.07 | 0.05 | 0.23 |
|  | min | 10 | 6.08 | 0.08 | 1 | 13.58 | 0.04 | 9 | 1.22 | 12 | 2.59 | 0.02 | 0.27 | 0.05 | 0.04 |
|  | max | 53 | 9.55 | 0.33 | 11 | 28 | 0.38 | 19 | 1.26 | 43 | 4.91 | 0.72 | 1.01 | 0.51 | 2.48 |
| **P18** | Mean | 48.11 | 6.28 | 0.3 | 1.56 | 26.67 | 27.44 | 6.2 | 1.77 | 306.89 | 3.37 | 0.9 | 1.23 | 0.05 | 2.43 |
|  | SE | 11.73 | 0.37 | 0.06 | 0.31 | 10.05 | 6.53 | 0.33 | 3.03 | 76.34 | 0.16 | 0.37 | 0.42 | 0.01 | 0.67 |
|  | min | 7 | 4.32 | 0.05 | 1 | 11.3 | 0.01 | 5 | 1.40 | 43 | 2.64 | 0.12 | 0.19 | 0.01 | 0.38 |
|  | max | 121 | 7.88 | 0.62 | 4 | 115.3 | 0.04 | 8 | 2.50 | 888 | 4.16 | 4.03 | 4.66 | 0.12 | 6.38 |
| **P19** | Mean | 10.4 | 4.98 | 0.22 | 1 | 18.31 | 0.26 | 14.1 | 1.91 | 55.4 | 2.88 | 0.27 | 0.74 | 0.33 | 0.6 |
|  | SE | 1.68 | 0.13 | 0.03 | 0 | 1.49 | 0.04 | 1.24 | 2.42 | 10.47 | 0.19 | 0.05 | 0.06 | 0.04 | 0.11 |
|  | min | 4 | 4.55 | 0.09 | 1 | 12.6 | 0.07 | 11 | 1.18 | 27 | 1.81 | 0.02 | 0.45 | 0.15 | 0.04 |
|  | max | 18 | 5.58 | 0.31 | 1 | 29.3 | 0.44 | 19 | 1.89 | 130 | 3.74 | 0.41 | 0.96 | 0.48 | 1.34 |

*N_vshoot_*, the number of vegetative shoots; *L_vshoot_*, the height of vegetative shoots; *M_vshoot_,* the weight of vegetative shoots; *N_rshoot_*, the number of reproductive shoots; *L_spike_*, the height of the longest spike; *M_rshoot_*, the weight of reproductive shoots; *N_seed_*, the number of seeds; *M_100seed_*, the weight of 100 seeds; *N_root_*, the number of roots; *L_root_*, the length of roots; *M_root_*, the total weight of roots; *M_total_*, the total biomass; *RVR*, reproductive biomass/vegetative biomass; *RSR*, underground biomass/aboveground biomass.
