# Supplementary material for: Morphological and Genetic Variation along a North-to-South Transect in Stipa purpurea, a Dominant Grass on the Qinghai-Tibetan Plateau: Implications for Response to Climate Change
Source: PLoS One. 2016 Aug 31;11(8):e0161972. doi: 10.1371/journal.pone.0161972 (PMC5006974; doi:10.1371/journal.pone.0161972)
Supplement: S4 Table — Bold-faced slopes indicate a significant deviation from 1. (DOCX) [file pone.0161972.s008.docx]

**S4 Table Estimates of slope (*b*, allometric exponent) and intercept (*a*, allometric coefficient) for *logR-logV* regressions within populations of *S. purpurea*. Bold-faced slopes indicate a significant deviation from 1**

| **Pop code** | ***R*^2^** | **Slope (*b*)** | **95%CI**  **(*b*)** | ***p*** | **vs. *b* = 1(H_0_)** |
| --- | --- | --- | --- | --- | --- |
| **P1** | 0.340 | **0.6115** | 0.384～0.974 | 0.039 | < |
| **P2** | 0.432 | 1.1358 | 0.553～2.331 | 0.717 | = |
| **P3** | 0.896 | -0.8295 | -1.745～-0.394 | 0.609 | = |
| **P4** | 0.364 | -1.4975 | -3.047～-0.736 | 0.250 | = |
| **P5** | 0.917 | 1.3450 | 0.639～2.831 | 0.419 | = |
| **P6** | 0.002 | **1.8073** | 1.176～2.777 | 0.011 | > |
| **P7** | 0.116 | 0.7948 | 0.417～1.516 | 0.462 | = |
| **P8** | 0.956 | 0.9818 | 0.367～2.624 | 0.969 | = |
| **P10** | 0.889 | -0.6888 | -1.660～-0.286 | 0.385 | = |
| **P11** | 0.220 | 1.2129 | 0.613～2.402 | 0.561 | = |
| **P12** | 0.057 | 0.5822 | 0.318～1.065 | 0.075 | = |
| **P13** | 0.312 | 1.3210 | 0.285～6.117 | 0.638 | = |
| **P14** | 0.015 | **2.9781** | 1.761～5.036 | 0.001 | > |
| **P15** | 0.019 | 0.9738 | 0.568～1.669 | 0.916 | = |
| **P17** | 0.274 | 1.7548 | 0.875～3.518 | 0.107 | = |
| **P18** | 0.375 | 0.9585 | 0.470～1.954 | 0.903 | = |
| **P19** | 0.282 | -1.0660 | -2.140～-0.531 | 0.850 | = |
