# Supplementary material for: Morphological and Genetic Variation along a North-to-South Transect in Stipa purpurea, a Dominant Grass on the Qinghai-Tibetan Plateau: Implications for Response to Climate Change
Source: PLoS One. 2016 Aug 31;11(8):e0161972. doi: 10.1371/journal.pone.0161972 (PMC5006974; doi:10.1371/journal.pone.0161972)
Supplement: S5 Table — Ne = ϑ/μ (μ = 10−3). (DOCX) [file pone.0161972.s009.docx]

**S5 Table Effective population size of *S. purpurea* estimated by MIGRATE**

| pop | *θ* | *Ne** |
| --- | --- | --- |
| P1 | 1.497 | 1497 |
| P2 | 1.187 | 1187 |
| P3 | 1.194 | 1194 |
| P4 | 1.214 | 1214 |
| P5 | 1.174 | 1174 |
| P6 | 1.088 | 1088 |
| P7 | 1.154 | 1154 |
| P8 | 1.211 | 1211 |
| P9 | 1.264 | 1264 |
| P10 | 1.206 | 1206 |
| P11 | 1.142 | 1142 |
| P12 | 1.242 | 1242 |
| P13 | 1.168 | 1168 |
| P14 | 1.198 | 1198 |
| P15 | 1.181 | 1181 |
| P16 | 1.083 | 1083 |
| P17 | 1.146 | 1146 |
| P18 | 1.240 | 1240 |
| P19 | 1.222 | 1222 |
| P20 | 1.227 | 1227 |
| Mean | 1.202 | 1202 |

**Ne* = *θ*/*μ* (*μ* = 10^-3^)
