# Supplementary material for: Morphological and Genetic Variation along a North-to-South Transect in Stipa purpurea, a Dominant Grass on the Qinghai-Tibetan Plateau: Implications for Response to Climate Change
Source: PLoS One. 2016 Aug 31;11(8):e0161972. doi: 10.1371/journal.pone.0161972 (PMC5006974; doi:10.1371/journal.pone.0161972)
Supplement: S6 Table — The migration rates are from the populations in the vertical row into the populations in the horizontal column, 95% confidence interval is shown in brackets. (DOCX) [file pone.0161972.s010.docx]

**S6 Table** **Estimates of bidirectional long-term gene flow (*M* = *m*/**μ**) between genetic regions using MIGRATE.**

|  | Qinghai Lake | Xidatan | Hoh Xil | Tanggula | Inland Tibet |
| --- | --- | --- | --- | --- | --- |
| Qinghai Lake | - | 0.612(0.424-0.843) | 0.310(0.186-0.481) | 0.185(0.093-0.323) | 0.339(0.205-0.518) |
| Xidatan | 0.206(0.119-0.327) | - | 0.822(0.634-1.045) | 0.810(0.619-1.036) | 0.727(0.549-0.941) |
| Hoh Xil | 0.027(0.003-0.096) | 1.194(0.951-1.474) | - | 1.117(0.874-1.410) | 0.721(0.532-0.954) |
| Tanggula | 0.365(0.239-0.529) | 1.213(0.963-1.497) | 0.924(0.707-1.186) | - | 0.880(0.673-1.125) |
| Inland Tibet | 0.387(0.243-0.577) | 1.357(1.062-1.701) | 1.613(1.286-1.986) | 0.998(0.750-1.298) | - |

The migration rates are from the populations in the vertical row into the populations in the horizontal column, 95% confidence interval is shown in brackets.
