# Supplementary material for: Morphological and Genetic Variation along a North-to-South Transect in Stipa purpurea, a Dominant Grass on the Qinghai-Tibetan Plateau: Implications for Response to Climate Change
Source: PLoS One. 2016 Aug 31;11(8):e0161972. doi: 10.1371/journal.pone.0161972 (PMC5006974; doi:10.1371/journal.pone.0161972)
Supplement: S7 Table — (DOCX) [file pone.0161972.s011.docx]

**S7 Table** **Estimates of** **bidirectional contemporary gene flow (*m*) between genetic regions using BAYESASS.**

|  | Qinghai Lake | Xidatan | Hoh Xil | Tanggula | Inland Tibet |
| --- | --- | --- | --- | --- | --- |
| Qinghai Lake | - | 0.0023(0.0023) | 0.0515(0.0112) | 0.0023(0.0022) | 0.0022(0.0022) |
| Xidatan | 0.0086(0.0056) | - | 0.0065(0.0053) | 0.0031(0.0031) | 0.0030(0.0030) |
| Hoh Xil | 0.0025(0.0024) | 0.0236(0.0067) | - | 0.0026(0.0024) | 0.0018(0.0018) |
| Tanggula | 0.0081(0.0062) | 0.0702(0.0183) | 0.1509(0.0194) | - | 0.0045(0.0044) |
| Inland Tibet | 0.0016(0.0016) | 0.0016(0.0016) | 0.1651(0.0113) | 0.0546(0.0084) | - |

The migration rates are from the populations in the vertical row into the populations in the horizontal column. Standard Deviation (SD) is shown in brackets.
